# Structural Extremes in a Cretaceous Dinosaur

**DOI:** 10.1371/journal.pone.0001230

**Published:** 2007-11-21

**Authors:** Paul C. Sereno, Jeffrey A. Wilson, Lawrence M. Witmer, John A. Whitlock, Abdoulaye Maga, Oumarou Ide, Timothy A. Rowe

**Affiliations:** 1 Department of Organismal Biology and Anatomy, University of Chicago, Chicago, Illinois, United States of America; 2 Museum of Paleontology and Department of Geological Sciences, University of Michigan, Ann Arbor, Michigan, United States of America; 3 Department of Biomedical Sciences, College of Osteopathic Medicine, Ohio University, Athens, Ohio, United States of America; 4 Institute for Human Science, University of Niamey, Niamey, Republic of Niger; 5 Jackson School of Geological Sciences, The University of Texas at Austin, Austin, Texas, United States of America; University of Oxford, United Kingdom

## Abstract

Fossils of the Early Cretaceous dinosaur, *Nigersaurus taqueti*, document for the first time the cranial anatomy of a rebbachisaurid sauropod. Its extreme adaptations for herbivory at ground-level challenge current hypotheses regarding feeding function and feeding strategy among diplodocoids, the larger clade of sauropods that includes *Nigersaurus*. We used high resolution computed tomography, stereolithography, and standard molding and casting techniques to reassemble the extremely fragile skull. Computed tomography also allowed us to render the first endocast for a sauropod preserving portions of the olfactory bulbs, cerebrum and inner ear, the latter permitting us to establish habitual head posture. To elucidate evidence of tooth wear and tooth replacement rate, we used photographic-casting techniques and crown thin sections, respectively. To reconstruct its 9-meter postcranial skeleton, we combined and size-adjusted multiple partial skeletons. Finally, we used maximum parsimony algorithms on character data to obtain the best estimate of phylogenetic relationships among diplodocoid sauropods. *Nigersaurus taqueti* shows extreme adaptations for a dinosaurian herbivore including a skull of extremely light construction, tooth batteries located at the distal end of the jaws, tooth replacement as fast as one per month, an expanded muzzle that faces directly toward the ground, and hollow presacral vertebral centra with more air sac space than bone by volume. A cranial endocast provides the first reasonably complete view of a sauropod brain including its small olfactory bulbs and cerebrum. Skeletal and dental evidence suggests that *Nigersaurus* was a ground-level herbivore that gathered and sliced relatively soft vegetation, the culmination of a low-browsing feeding strategy first established among diplodocoids during the Jurassic.

## Introduction

Some 50 years ago the first bones of an unusual sauropod, dubbed *Rebbachisaurus*, came to light in Upper Cretaceous rocks in Morocco [1]. In last twenty years, the fragile bones and slender teeth of close relatives have been recorded in Cretaceous rocks in Europe [2]–[4], South America [5]–[8] and elsewhere in Africa [9], [10]. The Rebbachisauridae is now understood as a far-reaching radiation of diplodocoid sauropods [4], [10], a group that also includes the Dicraeosauridae and the more familiar long-necked Diplodocidae. The majority of rebbachisaurid finds to date, nevertheless, are relatively incomplete, especially with regard to the skull.

Here we provide the first view of a relatively complete rebbachisaurid skull and skeleton pertaining to the African species *Nigersaurus taqueti* (Text S1, Figure S1, S2). The extremely delicate construction of the skull and axial column in *Nigersaurus* is counterintuitive for a terrestrial herbivore with an estimated body mass comparable to an elephant. An endocast reveals several features of a sauropod brain never before seen, and evidence from the inner ear shows that the muzzle points directly toward the ground. This habitual head posture is one of several extreme adaptations in *Nigersaurus* for ground-level browsing, a feeding strategy that may have played an important role in the diplodocoid radiation of the Jurassic.

## Results and Discussion

### Skull Morphology and Habitual Head Posture

**Figure 1:**
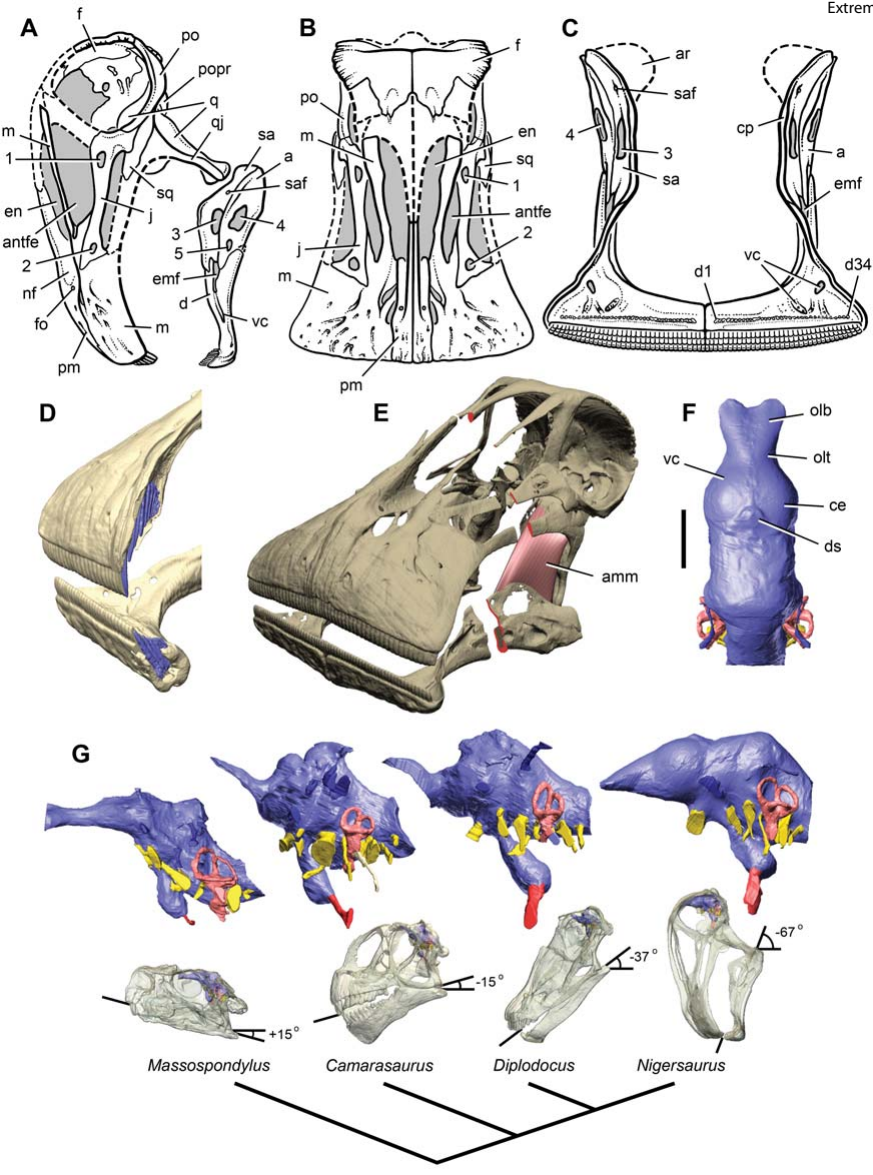
Skull of *Nigersaurus taqueti* and head posture in sauropodomorphs. (A)-Lateral view of skull (MNN GAD512). (B)-Anterodorsal view of cranium. (C)-Anterior view of lower jaws. (D)-Premaxillary and dentary tooth series (blue) reconstructed from µCT scans. (E)-Skull reconstruction in anterolateral and dorsal view cut at mid length between muzzle and occipital units (cross-section in red) with the adductor mandibulae muscle shown between the quadrate and surangular. (F)-Endocast in dorsal view showing the cerebrum and olfactory bulbs. (G)-Endocast (above) and transparent skulls with endocasts in place (below) based on µCT scans of the basal sauropodomorph *Massospondylus carinatus* (BP 1/4779), the basal neosauropod *Camarasaurus lentus* (CM 11338), the diplodocid *Diplodocus longus* (CM 11161), and the prototype skull of the rebbachisaurid *Nigersaurus taqueti*. Endocasts and skulls are oriented with the lateral semicircular canal held horizontal; the angle measurement indicates degrees from the horizontal for a line from the jaw joint to the tip of the upper teeth. Endocasts show brain space and dural sinuses (blue), nerve openings (yellow), inner ear (pink), and internal carotid artery (red). Phylogenetic diagram at bottom shows the relationships of these four sauropodomorphs, with increasing ventral deflection of the snout toward the condition seen in *Nigersaurus*. Scale bar equals 2 cm in F. *Abbreviations*: *1–5*, fenestrae 1–5; *a*, angular; *amm*, adductor mandibulae muscle; *antfe*, antorbital fenestra; *ar*, articular; *ce*, cerebrum; *cp*, coronoid process; *d*, dentary; *d1*, *34*, dentary tooth 1, 34; *ds*, dural sinus; *emf*, external mandibular fenestra; *en*, external nares; *f*, frontal; *fo*, foramen; *j*, jugal; *m*, maxilla; *nf*, narial fossa; *olb*, olfactory bulb; *olt*, olfactory tract; *pm*, premaxilla; *po*, postorbital; *popr*, paroccipital process; *q*, quadrate; *qj*, quadratojugal; *sa*, surangular, *saf*, surangular foramen; *sq*, squamosal; *vc*, vascular canal.

We used prototypes derived from high-resolution computed-tomography (µCT) scans of the bones of a single adult individual to recompose the extremely lightweight skull (Figure 1A–E). The delicate construction of the skull is well seen in transverse cross-section (Figure 1E, red), in which the total area of all bone connecting the muzzle to the occipital unit of the skull is approximately 1.0 cm^2^ (Text S2, Figure S3). These delicate connecting struts of bone, which rarely exceed 2 mm in thickness, must resist stress generated by the distally located tooth rows.

Several cranial features of *Nigersaurus* have never been reported in any other sauropodomorph dinosaur including closure of the supratemporal opening, five additional fenestrae on the lateral aspect of the skull, and teeth packed into terminal dental batteries that extend laterally beyond the side of the skull [10]. The external nares are large elongate openings that are only partially retracted, and the ends of the upper and lower jaws have numerous impressed neurovascular grooves suggestive of a keratinized sheath (Figure 1B, E).

The L-shaped lower jaw is divided into a subcylindrical transverse ramus housing the tooth battery and a lightweight posterior ramus, to which the principal jaw musculature attached (Figure 1C–E). Several novel openings, or fenestrae, are present in the lower jaw. Besides re-acquiring an external mandibular fenestra (which is closed in all other neosauropods)(Figure 1A), there are three other openings that are unknown elsewhere among sauropods, the largest centered on the thin, platelike surangular immediately underneath the attachment area for the jaw adductor musculature (Figure 1A, C, E). The adductor musculature, which normally inserts on the bony rim of the supratemporal fenestra, has a straight line of action to the coronoid process on the lower jaw. In *Nigersaurus*, however, that fenestra is closed and the line of action to the lower jaw blocked by a sharp bend in the quadrate. As the quadrate shows no sign of a synovial surface or trochlea to bend this line of action (as occurs in some turtles), the origin of the adductor musculature must have migrated onto the quadrate (Figure 1E, red muscle mass). Although we have no direct means to estimate the strength of the adductor musculature, the thin fenestrated structure of the surangular suggests that it was considerably weaker than in most other sauropods.

The jaw muscles that originate on the palate (pterygoideus musculature), likewise, appear to have been relatively weak. The elongate basipterygoid processes that buttress the palate against the pull of pterygoideus musculature taper distally to rods no more than 2 mm in diameter.

The head was habitually held with its muzzle pointing directly downward (Figures 1A, G (right skull), a striking pose that is rotated some 70 degrees from the usual (horizontal) anatomical depiction of a diplodocoid skull [11]. Habitual head posture was established on the basis of the lateral semicircular canal of the inner ear [12], [13], which was rendered from µCT scans. With the lateral semicircular canal positioned in a horizontal plane, the muzzle points downward, and the occipital condyle is exposed posteriorly for articulation with the cervical column (Figure 1A, G, Text S3, Figure S4, S5). Although downward rotation of the muzzle in some sauropods has been previously proposed [12], [14], [15], skull orientation based only on the anatomy of the occiput (foramen magnum, occipital condyle) also depends on the interpretation of neck posture; as the neck is inclined upward, the snout end of the skull also is raised. Here we present a specific orientation of the skull based on independent evidence from the inner ear. Compared to sauropodomorph outgroups, the muzzle is progressively rotated downward relative to the horizontal in diplodocoids, such as *Diplodocus* [16], and especially in *Nigersaurus* (Figure 1G).

### Anatomy and Function of a Tooth Battery

**Figure 2:**
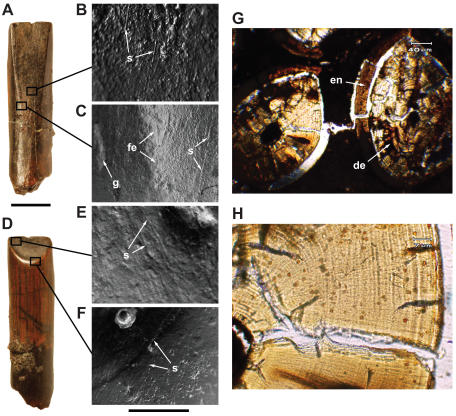
Crown form, wear pattern, and microstructure in *Nigersaurus taqueti*. Wear facets and surface detail is from a worn crown (MNN GAD513), and enamel and dentine microstructure is from left premaxillary teeth in cross-section (MNN GAD514). (A)-Crown in lingual (interior) view showing low-angle wear facet. Magnified views of a cast of the lingual wear facet showing (B)-wear striations in the dentine and (C)-the edge of the facet. (D)-Crown in labial (exterior) view showing high-angle wear facet. Magnified views of a cast of the labial facet showing (E)-coarse scratches on the dentine and (F)-fine scratches on the edge of the facet. (G)-Transverse thin section of two successive premaxillary crowns showing thickened labial enamel and circumferential incremental lines of von Ebner in the dentine. (H)-Magnified view of the older (left) crown showing approximately 60 incremental lines of von Ebner. Scale bar in A and D equals 5 mm; scale bar below F equals 0.5 mm in B, E and F and 1.11 mm in C. *Abbreviations*: *de*, dentine; *en*, enamel; *fe*, facet edge; *g*, gouge; *s*, scratch.

The premaxilla, maxilla and dentary are packed with slender teeth packed into opposing tooth batteries, the upper teeth slightly broader and less numerous than the lower and both with asymmetrical enamel that is approximately 10 times thicker on the labial side [2] (Figure 2G). We used µCT to reveal the internal packing of the battery and assist in creating an accurate digital reconstruction of a upper and lower replacement series (Figure 1D). In the center of upper and lower batteries, as many as 10 teeth are present in a single column extending deep within each jaw bone. The upper series has about 60 tooth columns (4 premaxillary, 25 maxillary per side), and the lower series has has 68 tooth columns (34 per dentary). In sum, there are more than 500 active and replacement teeth in a single skull.

As individual teeth migrate into an active position, tooth-to-tooth abrasion results in a low-angle, planar wear facet (ca. 5° from the long axis of the crown) on the lingual (internal) surface of the upper teeth, which is similar to that in advanced titanosaurs (Figure 2A). Light microscopy reveals predominantly axially-oriented fine scratches and some deeper gouges on both the planar surface of the facet (dentine) and on the adjacent enamel covering on the side of the crown (Figure 2B, C). These gouges appear similar to ‘plucking’ in mammalian prismatic enamel, which is generated from tooth-to-tooth contact. At present worn dentary teeth are not preserved. Such crowns would be expected to show an opposing labial (external) tooth-to-tooth wear facet.

A second high-angle wear facet (ca. 40° from the long axis of the crown) forms on the labial (external) surface of the upper teeth. This facet is D-shaped, rather than circular, owing to its intersection with the lingual (internal) facet (Figure 2D). The labial facet also has axially-oriented fine scratches in the dentine but is bordered by a raised, polished rim of thickened enamel. The form and surface detail of the labial facet, which is very similar to that in the diplodocoids *Dicraeosaurus* [17] and *Diplodocus* [18]–[20], suggests that it formed via sustained abrasion with relatively soft plant materials (Figure 2E, F) (Text S4).

We estimated tooth replacement rate from thin sections of an intact premaxillary tooth battery of a second individual that included successive crowns approaching an active position (Figure 2G, H). Incremental lines of von Ebner, which represent daily deposition of dentine [21], were visible in longitudinal and transverse sections, the first of such growth data for any sauropodomorph. The successive crowns differ by fewer than 30 lines, indicating a very rapid tooth replacement rate of approximately one month, or about twice as fast as that observed in hadrosaurid tooth batteries [21]. This corroborates a previous suggestion that tooth replacement rate and the number of teeth in a single tooth column may be correlated [21], both of which are accelerated in *Nigersaurus* as compared to an average hadrosaurid (Text S4).

### An Endocast with Olfactory Bulbs and Cerebrum

Brain features were studied via cranial endocasts taken from the exceptionally preserved braincase using both traditional silicone peels and volumetric rendering from µCT scans (Figure 1F, G-right endocast, Text S3, Figure S4, S5). Only the parietal and supraoccipital surfaces of the endocast were reconstructed (because these bones are not preserved), a portion of the endocast that usually also accommodates venous sinuses surrounding the hindbrain [12]. The dural venous sinus in sauropods often expands anterodorsally into the frontal bone, obscuring the dorsal shape of the forebrain (Figure 1G, middle pair of endocasts). In *Nigersaurus*, however, the dural sinus is small, allowing the clearest view to date of the anterior end of a sauropod brain, including the olfactory bulbs, cerebrum, and cerebellar flocculus [12], [16] (Figure 1F, G-right endocast, Figure S4). The convex cerebrum in *Nigersaurus* arches above adjacent endocranial surfaces and comprises approximately 30% of total brain volume as in many dinosaurs such as *Tyrannosaurus* [22] (Figure 1F, Figure S4). An impressed vessel track crossing the hollow for the cerebrum provides evidence that the forebrain effectively filled the anterior endocranial space in sauropods as has been shown in other dinosaurs. The olfactory tracts are particularly short and the olfactory bulbs are small. Olfaction, thus, may have been less important behaviorally, despite the presence of a large fleshy narial region. Volumetric estimates from the endocast are 2.9, 16.6, and 53.4 cm^3^ for the paired olfactory bulbs, cerebrum, and total brain, respectively (Text S3, Figure S6). Total brain volume plots within the 95% confidence limits of a log regression of brain volume and estimated body mass in nonavian reptiles [22]. Both cerebral and total brain volume in *Nigersaurus*, nonetheless, are absolutely small relative to ornithischian and non-coelurosaurian theropods of comparable body size [12], measuring for example less than one-third that in the large basal tetanuran theropod *Carcharodontosaurus* [22].

### Skeletal Reconstruction

**Figure 3:**
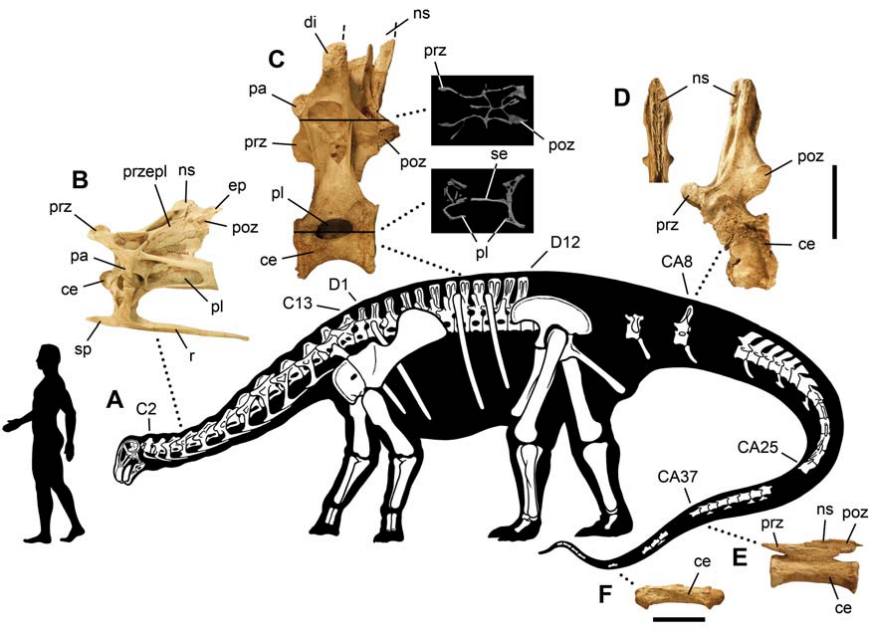
Skeleton of *Nigersaurus taqueti*. Skeletal reconstruction is based mainly on four specimens (MNN GAD513, GAD 515-518). (A)-Skeletal silhouette showing preserved bones. (B)-Fifth cervical vertebra in lateral view. (C)-Eighth dorsal vertebra in lateral view with two cross-sections from a µCT scan. (D)-Probable eighth caudal vertebra in lateral view with anterior view of the neural spine. (E)-Caudal vertebra (ca. CA37) with low neural spine. (F)-Distal caudal vertebra (ca. CA47) with biconvex centrum and rudimentary neural arch. Human silhouette equals 1.68 meters (5 feet 6 inches). Upper scale bar equals 10 cm for B-E; lower scale bar equals 5 cm for F. *Abbreviations*: *C*, cervical vertebra; *CA*, caudal vertebra; *ce*, centrum; *D*, dorsal vertebra; *di*, diapophysis; *ep*, epipophysis; *ns*, neural spine; *pa*, parapophysis; *pl*, pleurocoel; *poz*, postzygapophysis; *prz*, prezygapophysis; *przepl*, prezygapophyseal-epipophyseal lamina; *r*, rib; *se*, septum; *sp*, spine.

*Nigersaurus* is a relatively small sauropod with a femur length of 1 meter, a short cervical series (only 130% of the dorsal series) composed of only 13 vertebrae, and an adult body length of only approximately 9 m (Figure 3A). The first, and only previous, reconstruction of a rebbachisaurid, *Limaysaurus* [5], included a longer cervical series of 16 vertebrae, its proportions (160% of the dorsal series) and high vertebral count ostensibly based on diplodocids. As only eight cervical vertebrae are known, however, the relative length and vertebral count of the cervical series in rebbachisaurids remains an open question, one that has important ramifications for the basal diplodocoid condition.

**Figure 4:**
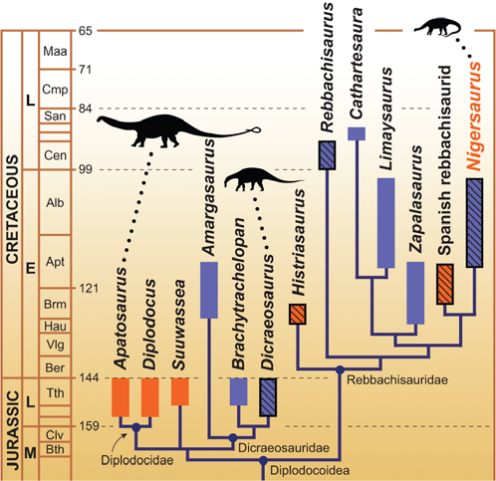
Calibrated phylogeny of diplodocoid sauropods. The diagram is based on strict consensus of five minimum-length trees using 13 ingroup taxa and 102 unordered characters (CI = 0.76; RI = 0.78) (Text S5). Scaled icons represent a diplodocid (*Apatosaurus*) [11], dicraeosaurid (*Dicraeosaurus*) [51], and a rebbachisaurid (*Nigersaurus*). Geographic distributions include Laurasian diplodocoids (western North America—*Apatasaurus*, *Diplodocus*, *Suuwassea*; Europe—*Histriasaurus*, Spanish rebbachisaurid) and Gondwanan diplodocoids (South America—*Cathartesaura, Limaysaurus, Zapalasaurus*; Africa—*Rebbachisaurus, Nigersaurus*). Temporal boundaries based on a recent timescale [52]. Color scheme: Laurasia (*orange*); Gondwana (*blue*); North America (*solid orange*); Europe (*striped orange*); South America (*blue*); Africa (*striped blue*).

A proportionately short neck (130% or less of the dorsal series) and modest body length of 10 meters or less characterizes nearly all rebbachisaurids [3]–[10] and dicraeosaurids [17], [23], [24] in contrast to the longer neck and larger body size in diplodocids (Figure 4, scaled body icons). In *Nigersaurus* the relatively short neck length and cervical vertebral count of 13 is established on the basis of two overlapping individuals that together compose a complete presacral series from the axis to the mid dorsal vertebrae. A relatively short neck of 13 vertebrae as in *Nigersaurus* is certainly equally plausible as the basal diplodocoid condition as opposed to the comparatively long-necked diplodocids. Regarding body size, only the poorly known *Rebbachisaurus* [1] is comparable to the larger-bodied diplodocids *Diplodocus* and *Apatosaurus*, which grow to twice the length and four times the estimated body mass of *Nigersaurus* [25]. Thus increase in cervical number, neck length, and body size may ultimately be understood as derived diplodocid features within Diplodocoidea. At present an alternative hypothesis that invokes parallel evolution of the reversed conditions in rebbachisaurids and dicraeosaurids cannot be definitively excluded until the position of other small-bodied basal diplodocoids, such as *Suuwassea*, are better resolved.

Pneumatic invasion of the centra of the presacral vertebrae has reduced the vertebral body to a hollow shell divided by a thin median septum (Figure 3C, lower cross-section), an extreme version of the condition in other diplodocoids [26], [27]. Internal cancellous bone is nearly eliminated; even the articular ends of the centra are little more than thin plates of bone. External pneumatic diverticulae, in turn, have reduced the dorsal neural arches to a set of intersecting laminae, which often are less than 2mm thick (Figure 3C, upper cross-section). The tail is the only part of the axial column that has solid centra. Anterior caudal vertebrae have cruciate neural spines with a characteristic flaring of the lateral lamina at mid length (Figure 3D, left), mid caudal vertebrae have proportionately low neural spines (Figure 3E), and distal caudal vertebrae have biconvex centra of moderate length (Figure 3F). Much of the pelvic and pectoral girdles are also reduced to thin sheets of bone several millimeters in thickness, including most of the paddle-shaped blade of the scapula and semicircular blade of the ilium (Figure 3A). The limb bones are less specialized and have diplodocoid proportions, the forelimb only 66% of hind limb length.

### A Featherweight Skull

*Nigersaurus* forces a consideration of the biomechanical limits of skull and axial design for a large-bodied, land-based herbivore and impacts our understanding of several aspects of sauropod paleobiology and evolution. No other herbivorous tetrapod has evolved a skull of comparable size with as little bone that is able to withstand sustained impact from tooth-to-tooth shearing. The shearing action of its jaws, furthermore, ground down crowns at a faster rate than ever recorded among dinosaurian herbivores and accomplished this dental degradation in the most challenging location from a biomechanical perspective—at the distal end of its jaws, as far as possible from the jaw joint and adductor musculature (Figure 1E). No tetrapod has ever allocated all of their teeth to such a distal position, much less doing so with an elaborate tooth battery. Somehow this masticatory apparatus was able to grow and maintain a body mass grossly commensurate with that of an average elephant (ca. 4 tons).

*Nigersaurus* does not incorporate any of the progressive modifications in jaw structure and musculature that arose in parallel during the evolution of dental batteries in ceratopsids and hadrosaurids or the elaborated masticatory apparatus of mammals. These include closure of fenestrae on the snout and lower jaw to solidify the posterior skull, enlargement of the dentary and development of a coronoid process for the direct and enhanced attachment of adductor musculature, and posterior displacement of teeth toward the adductor musculature and jaw hinge to increase their biomechanical advantage during mastication (Figure 1E). Although other diplodocids also have relatively gracile cranial design, *Nigersaurus* is more divergent in these regards. *Diplodocus*, for example, has a nearly solid snout wall with small retracted external nares, lower jaw without openings, adductor musculature that can be reconstructed with the usual origin and insertion, tooth rows with at least some teeth in parasagittal position, and tooth wear without tooth-to-tooth contact (Figure 1G, third skull to right). Bite force in *Nigersaurus*, we must conclude, must have been reduced compared to other sauropods including *Diplodocus*.

The skull of *Nigersaurus* presents an excellent case to test the utility of finite-element modeling of cranial form [28]. This method has been applied to skull shape in *Diplodocus* [29], but the resulting theoretical construction has far greater bone thickness than in *Diplodocus* and especially *Nigersaurus*. If optimal skull design is defined as “maximum strength with minimum material” [29], the skull of *Nigersaurus* is the outstanding case to evaluate.

### Evolution of Habitual Head Posture

Evidence from the inner ear highlights a profound reorientation of habitual head posture from the slightly upturned muzzle in the basal sauropodomorph *Massospondylus* to the progressively down-turned muzzle in the basal neosauropod *Camarasaurus*, the diplodocid *Diplodocus*, and the rebbachisaurid *Nigersaurus* (Figure 1G). The degree of downward rotation doubles from *Diplodocus* to *Nigersaurus* with concomitant increase in the width of the squared anterior margin of the muzzle.

Although the downward inclination of the skull of *Diplodocus* relative to the cervical series was noted in the nineteenth century shortly after its discovery [30] and discussed again when the first skeleton was mounted [31], the skull is sometimes oriented erroneously in recent studies with its long axis nearly aligned with that of the cervical column [e.g. 26]. As in other diplodocoids, the position of the foramen magnum and basioccipital condyle in *Nigersaurus* is aligned for articulation with the atlas only when the long axis of the skull is rotated so the muzzle points downward (Figure 3A).

Recent work on the neutral pose of the cervical column in diplodocids and dicraeosaurids also has strongly favored a horizontal or anteroventrally sloping inclination for the neck [14], [15], [32] as depicted long ago when the cervical series of *Diplodocus* was first described [33]. Osteological evidence for a strong upward inclination of cervical column for high browsing [34] is lacking. Independent evidence for strong downward deflection of the muzzle is provided here from the lateral semicircular canal of the inner ear. In sum, osteological evidence from the occiput, inner ear, and cervical vertebrae in both *Diplodocus* and *Nigersaurus* favors an habitual, or neutral, posture for head and neck with the muzzle rotated downward not far from ground level (Figure 3A).

### Feeding Function

The limited capability of the cervical vertebrae for dorsiflexion probably restricted diplodocoids to browsing near the ground. Models estimating maximum cervical dorsiflexion have given a browsing range of up to 4 and 6 meters for the diplodocids *Diplodocus* and *Apatosaurus*, respectively [32]. Others have estimated a potential browsing range for diplodocids up to 10 meters or more [35]. The shorter neck of dicraeosaurids and rebbachisaurids would have been far more limiting in this regard. The ability to raise the head to any height, of course, does not necessarily mean that these sauropods regularly sought browse many meters above the ground.

Here we distinguish *ground-level browsing* as a specialized form of low browsing that involves the gathering and cropping of plants close to the ground. Ground-level browsing may be understood as the Mesozoic functional parallel of grazing among mammalian herbivores of the Cenozoic. Low-lying plants or ground cover, of course, did not include grass until the latest Cretaceous but rather consisted of ferns, horsetails and other non-angiosperms during the Jurassic and most of the Early Cretaceous and possibly some angiosperms by mid Cretaceous times (Aptian-Albian) when *Nigersaurus* lived. Previous reference to “head down” feeding by “ventriflexion” [15] included both browsing near the ground as well as subsurface aquatic feeding by straining. Here we use ‘ground-level browsing’ only for the gathering and cropping of browse within one meter or less of the soil surface.

Squaring of the muzzle in diplodocoids in general and *Nigersaurus* in particular suggests increasing adaptation to ground-level browsing. Broadening of the muzzle [36] or premaxilla [37] have proven to be excellent indicators of grazing versus mixed feeding or browsing in many different groups of extant mammalian herbivores. The extraordinary breadth of the muzzle of *Nigersaurus* and the squared muzzles in other diplodocoids would enhance gathering and cropping near a relatively flat surface.

Unusual wear facets on the labial (external) aspect of the dentition in *Nigersaurus* (Figure 2D) are also present in dicraeosaurids [17] and diplodocids [18]. If additional work confirms these as similar in orientation and microwear, they may be indicative of a similar, or at least overlapping, feeding strategy among diploocoids. We suggest here that this enigmatic, external, high-angle facet with polished enamel edge may have formed from tooth-to-plant abrasion occurring during ground-level browsing.

The spectrum of previous explanations for tooth form and wear in diplodocoids include molluscivory or piscivory [18], straining and cropping aquatic plants [18], [38], straining planktonic plants and animals [39], bark stripping [18], [40], and low and high-level leaf-stripping or raking [18], [20], [38], [41], [42]. None of these explanations account for the squared muzzle or strong declination of the snout away from the axis of the cervical column. These features are difficult to understand either as adaptations to bark- or leaf-stripping or to aquatic feeding. Rather they function well in placing the muzzle close to the ground. In *Nigersaurus*, in particular, the snout could not have been directed anteriorly to strip leaves or generate an external wear facet without disarticulation of the skull from the neck. Estimating ‘feeding envelopes’ based on cervical vertebrae is revealing [15], [32] but may have even greater functional significance when the potential range of motion of the skull is considered simultaneously.

Finally, jaw movement in dicraeosaurids and diplodocids has long been interpreted as propalinal (fore-aft), either with [43] or without [19], [20], [41], [42] tooth-to-tooth occlusion. Given the evidence from *Nigersaurus*, this interpretation is open to question. Although the dentition in *Diplodocus* and some diplodocoids is less compact and more prognathous than *Nigersaurus*, there are several fundamental similarities, including the distal position of most of the tooth row in a squared jaws, slender crown shape, and increased rate of tooth replacement (judging from the number of crowns in the replacement series). Like *Diplodocus* and *Dicraeosaurus*, *Nigersaurus* has a high-angle labial (external) wear facet, the product of abrasion against plant or other materials (Figure 2D). *Nigersaurus*, however, also has a low-angle, tooth-to-tooth wear facet on the inside of the maxillary crowns (Figure 2A) that provides indisputable evidence for a precisely controlled, orthal jaw stroke. Orthal and propalinal jaw motion cannot plausibly coexist and create the regular pattern of intersecting facets observed on individual crowns in *Nigersaurus*. We tentatively interpret the labial (external) facet in *Nigersaurus* and possibly other diplodocoids, thus, as evidence of tooth-to-plant abrasion that occurred as vegetation was gathered near the ground prior to orthal shearing.

In sum, although the paradigm for feeding among diplodocoids has shifted from high browsing [34] to low browsing by leaf-stripping across a range as high as to 4–10 meters [15], [19], [20], [32], [41], [42], it appears that ground-level browsing may constitute a significant, even dominant, feeding strategy among these sauropods. Habitual head posture in *Diplodocus* and *Nigersaurus* and constraints on forward rotation of the skull seem to favor browsing near the ground, which would have lowered and diversified the ‘browsing profile’ of faunas with several sauropods [44]. More detail is needed on diplodocoid dentitions to reach firm functional conclusions regarding these intriguing herbivores.

### Plant Resources

The identity of specific plant groups as food resources for *Nigersaurus* and other diplodocoids is speculative due to the paucity of evidence from stomach or fecal contents, the true composition of local flora, and the poor correlation between tooth form/wear and plant type. Nevertheless, the weak bite force and fine scale of scratches on the wear facets in *Nigersaurus* favor soft understory vegetation, possibly immature ferns or horsetails [45], an interpretation that is at least consistent with a mid Cretaceous (ca. 110 Ma) habitat dominated by inland floodplains associated with high-energy river systems. Any hypothetical plant resource must have been fairly widespread, as *Nigersaurus* and the iguanodontian *Lurdusaurus* [46] were the most common megaherbivores of the day.

Conifers, cycads, aquatic vegetation, and grass are less likely primary plant resources for *Nigersaurus* and other diplodocoids due to their height, resistant structure, absence of a suitable local habitat, and late appearance in the fossil record, respectively. Aquatic vegetation is difficult to imagine as a primary resource in the varied habitats that have yielded diplodocoid remains. Cross-bedded sandstones, rather than sediments indicative of still water, comprise nearly all of the Elrhaz Formation, where the bones of *Nigersaurus* are found (Text S1). Seasonally dry habitats are often the favored interpretation for the Morrison Formation and other localities where diplodocoids are abundant. Likewise, grass does not register in the fossil record until the latest Cretaceous [47], whereas diplodocoids had evolved the squared jaw and dental specializations before the close of the Jurassic. Despite some concerns regarding caloric content [48], ferns and horsetails are geographically widespread, grow well in a wide variety of habitats, and may be the most probable primary plant resource for many diplodocoids.

### Diplodocoid Phylogeny

When *Nigersaurus* is placed in phylogenetic context (Figure 4), it shares its closest relationship with a recently described Spanish rebbachisaurid [4]. This trans-Tethyan link between Africa and Europe during the Cretaceous is a paleobiogeographic pattern that is also found among contemporary spinosaurid dinosaurs and several other vertebrate groups [49] and appears be the result of persistent connections across carbonate platforms in the Tethyan seaway [50].

Diplodocids, which flourished in Upper Jurassic deposits of western North America, represent an older Jurassic split among diplodocoids. These substantially larger-bodied, longer-necked genera, which include the familiar genus *Diplodocus,* have long served as the model for an increasingly diverse Diplodocoidea. The discovery of *Nigersaurus* and other rebbachisaurids and dicraeosaurids now leaves open the basal condition for Diplodocoidea (Figure 4). It is possible that early diplodocoids had a proportionately short neck (130% or less of back length), down-turned muzzle, and possibly also a modest body size (10 meters or less). They now appear to have radiated across both Gondwana and Laurasia during the Cretaceous quite possibly as ground-level browsers. This hypothesis stands to be tested as new fossils with more complete dentitions come to light.

### Conclusions

The skull of the sauropod *Nigersaurus taqueti* provides a striking case of minimal structural material for a large-bodied herbivore comparable in body mass to an elephant. Despite its lightweight design, the skull had an extremely active dentition that produced tooth-to-tooth wear facets at the distal end of transversely broadened jaws.

The expanded muzzle faces directly toward the ground, a habitual head posture determined here on the basis of the lateral semicircular canal of the inner ear. The unusual labial (external) abrasion facet on its teeth may have been generated during ground-level browsing. The low-angle, planar facet on in the inside of the maxillary crowns indicates that *Nigersaurus* had an orthal chewing cycle involving tooth-to-tooth contact.

Because other diplodocoids share with *Nigersaurus* many cranial and dental features including a squared end to the lower jaw and a labial abrasion facet, they may have shared aspects of their feeding strategies. It seems plausible, at the very least, that other diplodocoids had orthal, rather than a propalinal (fore-aft), jaw movement.

Modest body length, a proportionately short neck, and downward deflection of the muzzle in *Nigersaurus* are conditions that characterize many diplodocoids and now represent an alternative basal condition for the clade. *Nigersaurus* is the culmination of a low-browsing feeding strategy among diplodocoids that originated in the mid Jurassic and may have had an ecologically significant impact on surface vegetation on several land areas during the Cretaceous.

## Supporting Information

Figure S1 Partial skeleton of *Nigersaurus taqueti* (MNN GAD517) discovered during the 2000 Expedition to Niger. Expedition member G. Lyon is seated inside the curve of the proximal caudal vertebrae of a skeleton planed flat by wind-blown sand at a site in Gadoufaoua, Ténéré Desert, Niger (photo by M. Hettwer).
(0.34 MB TIF)

Figure S2 Location of outcrops of the Elrhaz Formation where fossils of *Nigersaurus taqueti* were found.
(0.13 MB TIF)

Figure S3 Assembled semi-translucent skull model of *Nigersaurus taqueti* built from prototyped skull bones (tooth batteries and reconstructed teeth in red) with unknown bones in green modeling clay (photo by T. Keillor).
(3.47 MB TIF)

Figure S4 Cranial endocast, endosseous labyrinth, and some endocranial vascular structures in *Nigersaurus taqueti* (MNN GAD512) derived from surface renderings of µCT scan data. (A)-left lateral view. (B)-left anteroventrolateral view. (C, H)-ventral view. (D, I)-dorsal view. (E, J)-anterior view. (F, K)-posterior view. *Color scheme*: *cyan blue*, cranial endocast; *pink*, endosseous labyrinth; *yellow*, nerve canals (some of which also transmit veins); *red*, arterial canals; *dark blue*, smaller venous canals. *Abbreviations*: *car*, cerebral carotid artery canal; *cer*, cerebral hemisphere; *cvcm*, caudal middle cerebral vein; *de*, dural expansion; *fl*, flocculus ( = cerebellar auricle); *lab*, endosseous labyrinth; *ob*, olfactory bulb; *ocv*, orbitocerebral vein; *opt?*, possible optic tectum ( = lobe); *pfo*, pituitary ( = hypophyseal) fossa; *II*, optic nerve canal; *III*, oculomotor nerve canal; *IV*, trochlear nerve canal; *V*, trigeminal nerve canal; *VI*, abducens nerve canal; *VII*, facial nerve canal; *VIII*, canal for vestibular branch of vestibulocochlear nerve; *IX-XI*, shared canal for glossopharyngeal, vagus, and accessory nerves and accompanying vessels; *XII*, hypoglossal canal.
(7.88 MB TIF)

Figure S5 Endosseous labyrinths of the left inner ear of (A-C, stereopairs) the rebbachisaurid *Nigersaurus taqueti* (MNN GAD512), (D-E) the diplodocid *Diplodocus longus* (CM 11161), and (F-G) the basal neosauropod *Camarasaurus lentus* (CM 11338). (A, D, F)-left lateral view. (B, F, G)-dorsal view. (C)-posterior view. *Abbreviations*: *c*, cochlea; *csc*, caudal (posterior vertical) semicircular canal; *fc*, fenestra cochlea ( = round window); *fv*, fenestra vestibuli ( = oval window); *lsc*, lateral (horizontal) semicircular canal; *rsc*, rostral (anterior vertical) semicircular canal; *ve*, vestibule of inner ear.
(2.41 MB TIF)

Figure S6 Partitioned endocast with transparent osseous labyrinth from *Nigersaurus taqueti*. Colors highlight the partitions used for digital assessment of endocast volumes.
(1.27 MB TIF)

Text S1 Geologic setting.
(0.07 MB PDF)

Text S2 The prototype skull.
(0.08 MB PDF)

Text S3 Endocast and labyrinth.
(0.15 MB PDF)

Text S4 Microwear and incremental lines of von Ebner.
(0.13 MB PDF)

Text S5 Phylogenetic analysis.
(0.16 MB PDF)

## References

[1] Lavocat R (1954). Sure les dinosauriens du Continental Intercalaire des Kem-Kem de la Daoura.. Comptes Rendus de la Dix-Neuviéme Session, Congrès Géologique International, Alger.

[2] Naish D, Martill DM, Martill DM, Naish D (2001). Saurischian dinosaurs 1: Sauropods.. Dinosaurs of the Isle of Wight.

[3] Dalla Vecchia FM (1998). Remains of Sauropoda (Reptilia, Saurischia) in the Lower Cretaceous (upper Hauterivianl/lower Baremian) limestones of SW Istria (Croatia).. Geol Croatica.

[4] Pereda-Suberbiola X, Torcida F, Izquierdo LA, Huerta P, Montero D (2003). First rebbachisaurid dinosaur (Sauropoda, Diplodocoidea) from the Early Cretaceous of Spain: paleobiological implications.. Bull Soc Géol France.

[5] Calvo JO, Salgado L (1995). *Rebbachisaurus tessonei* sp. nov. a new Sauropoda from the Albian-Cenomanian of Argentina; new evidence on the origin of Diplodocidae.. Gaia.

[6] Salgado L, Garrido A, Cocca SE, Cocca JR (2004). Lower Cretaceous rebbachisaurid sauropods from Cerro Aguada Del León (Lohan Cura Formation), Neuquén Province Northwestern Patagonia. Argentina.. J Vert Paleont.

[7] Gallina PA, Apesteguía S (2005). *Cathartesaura anaerobica* gen. et sp. nov., a new rebbachisaurid (Dinosauria, Sauropoda) from the Huincul Formation (Upper Cretaceous), Río Negro, Argentina.. Rev Mus Argentino Cienc Nat, n s.

[8] Kellner AWA (1996). Remarks on Brazilian dinosaurs.. Memoirs of the Queensland Museum.

[9] Sereno PC, Beck AL, Dutheil DB, Larsson HC, Lyon GH (1999). Cretaceous sauropods from the Sahara and the uneven rate of skeletal evolution among dinosaurs.. Science.

[10] Sereno PC, Wilson JA, Curry Rogers KA, Wilson JA (2005). Structure and evolution of a sauropod tooth battery.. The Sauropods: Evolution and Paleobiology.

[11] Wilson JA, Sereno PC (1998). Early evolution and higher-level phylogeny of sauropod dinosaurs.. J Vert Paleontol Mem 7.

[12] Hopson JA, Gans C (1979). Paleoneurology.. Biology of the Reptilia.

[13] Witmer LM, Chatterjee S, Franzosa J, Rowe T (2003). Neuroanatomy of flying reptiles and implications for flight, posture and behaviour.. Nature.

[14] Martin J, Currie PM, Koster EH (1987). Mobility and feeding of *Cetiosaurus* (Saurischia, Sauropoda)—why the long neck?. Fourth Symposium on Mesozoic Terrestrial Ecosystems, Short Papers: Occasional Papers of the Tyrell Museum of Palaeontology.

[15] Stevens KA, Parrish JM, Curry Rogers KA, Wilson JA (2005a). Digital reconstructions of sauropod dinosaurs and implications for feeding.. The Sauropods: Evolution and Paleobiology.

[16] Witmer LM, Ridgely RC, Dufeau DL, Semones MC, Frey R, Endo H (in press). Using CT to peer into the past: 3D visualization of the brain and ear regions of birds, crocodiles, and nonavian dinosaurs.. Anatomical Imaging: Towards a New Morphology.

[17] Janensch W (1929). Die wirbelsäule der gattung *Dicraeosaurus*.. Palaeontogr (Suppl 7).

[18] Holland WJ (1924). The skull of *Diplodocus*.. Mem Carnegie Mus.

[19] Fiorillo AR (1998). Dental microwear patterns of the sauropod dinosaurs *Camarasaurus* and *Diplodocus*: evidence for resource partitioning in the Late Jurassic of North America.. Hist Biol.

[20] Upchurch P, Barrett PM, Sues H-D (2000). The evolution of sauropod feeding mechanisms.. Evolution of Herbivory in Terrestrial Vertebrates: Perspectives from the Fossil Record.

[21] Erickson GM (1996). Incremental lines of von Ebner in dinosaurs and the assessment of tooth replacement rates using growth line counts.. Proc Natl Acad Sci USA.

[22] Larsson HCE, Sereno PC, Wilson JA (2000). Forebrain enlargement among nonavian theropod dinosaurs.. J Vert Paleont.

[23] Salgado L, Bonaparte JF (1991). Un nuevo sauropodo Dicraeosauridae, *Amargasaurus casaui* gen. et sp. nov., de la fomación La Amarga, Neocomiano de la Provincia del Neuquén, Argentina.. Ameghiniana.

[24] Rauhut OWM, Remes K, Fechner R, Cladera G, Puerta P (2005). A remarkably short-necked sauropod dinosaur from the Late Jurassic of Patagonia.. Nature.

[25] Seebacher F (2001). A new method to calculate allometric length-mass relationships of dinosaurs.. J Vert Paleont.

[26] Schwarz D, Frey E, Meyer CA (2007). Pneumaticity and soft−tissue reconstructions in the neck of diplodocid and dicraeosaurid sauropods.. Acta Palaeontol Pol.

[27] Wedel MJ, Curry Rogers KA, Wilson JA (2005). Postcranial skeletal pneumaticity in sauropods and its implications for mass estimates.. The Sauropods: Evolution and Paleobiology.

[28] Ross C (2005). Finite element analysis in vertebrate biomechanics.. Anat Rec.

[29] Witzel U, Preuschoft H (2005). Finite-element model construction for the virtual synthesis of the skulls in vertebrates: case study of *Diplodocus*.. Anat Rec.

[30] Marsh OC (1896). Dinosaurs of North America.. Ann Rep, U S Geol Surv, Pt 1.

[31] Holland WJ (1906). The osteology of *Diplodocus* Marsh.. Mem Carnegie Mus.

[32] Stevens KA, Parrish JM (1999). Neck posture and feeding habits of two Jurassic sauropod dinosaurs.. Science.

[33] Hatcher JB (1901). *Diplodocus* (Marsh): Its osteology, taxonomy, and probable habits, with a restoration of the skeleton.. Memoirs of the Carnegie Museum.

[34] Barrett PM, Willis KJ (2001). Did dinosaurs invent flowers? Dinosaur-angiosperm coevolution revisited.. Biological Reviews.

[35] Bakker RT (1978). Dinosaur feeding behavior and the origin of flowering plants.. Nature.

[36] Janis CM, Ehrhardt D (1988). Correlation of relative muzzle width and relative incisor width with dietary preference in ungulates.. Zool J Linn Soc.

[37] Solounias N, Moelleken SMC (1993). Dietary adaptation of some extinct ruminants determined by premaxillary shape.. Journal of Mammalogy.

[38] Dodson P, Weishampel DB, Dodson P, Osmólska H (1990). Sauropod Paleoecology.. The Dinosauria. 1st ed.

[39] Stevens KA, Parrish JM, Tidwell V, Carpenter K (2005b). Neck posture, dentition, and feeding strategies in Jurassic sauropod dinosaurs.. Thunder-lizards: The Sauropodomorph Dinosaurs.

[40] Bakker RT (1986). The Dinosaur Heresies..

[41] Barrett PM, Upchurch P (1994). Feeding mechanisms of *Diplodocus*.. GAIA.

[42] Barrett PM, Upchurch P, Sun A, Wang Y (1995). Sauropod feeding mechanisms.. Sixth Symposium on Mesozoic Terrestrial Ecosystems and Biota, Short Papers.

[43] Calvo JO (1994). Jaw mechanics in sauropod dinosaurs.. GAIA.

[44] Coe MJ, Dilcher DL, Farlow JO, Jarzen DM, Russell DA, Friis EM, Chaloner WG, Crane PR (1987). Dinosaurs and land plants.. The Origins of Angiosperms and their Biological Consequences.

[45] Krassilov VA (1981). Changes of Mesozoic vegetation and the extinction of the dinosaurs.. Palaeogeogr, Palaeoclimatol, Palaeoecol.

[46] Taquet P, Russell DA (1999). A massively-constructed iguanodont from Gadoufaoua, Lower Cretaceous of Niger.. Ann Paleontol.

[47] Prasad V, Strömberg CAE, Alimohammadian H, Sahni A (2005). Dinosaur coprolites and the early evolution of grasses and grazers.. Science.

[48] Weaver JC (1983). The improbable endotherm: The energetics of the sauropod dinosaur *Brachiosaurus*.. Paleobiol.

[49] Gheerbrant E, Rage JC (2006). Paleobiogeography of Africa: How distinct from Gondwana and Laurasia?. Palaeogeogr Palaeoclimatol Palaeoecol.

[50] Dalla Vecchia FM, Tidwell V, Carpenter K (2005). Between Gondwana and Laurasia: Cretaceous sauropods in an intraoceanic platform.. Thunder-lizards: The Sauropodomorph Dinosaurs.

[51] Wilson JA (2002). Sauropod dinosaur phylogeny: critique and cladistic analysis.. Zool J Linn Soc.

[52] Gradstein FM, Ogg JG, Smith AG (2004). A Geologic Time Scale 2004..

