# Supplementary figures and images for: Structural Extremes in a Cretaceous Dinosaur
Source: PLoS One. 2007 Nov 21;2(11):e1230. doi: 10.1371/journal.pone.0001230 (PMC2077925; doi:10.1371/journal.pone.0001230)

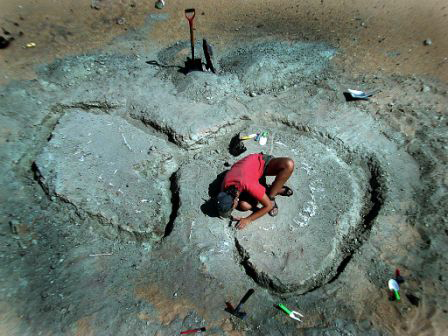

Supplement: Figure S1 — Partial skeleton of Nigersaurus taqueti (MNN GAD517) discovered during the 2000 Expedition to Niger. Expedition member G. Lyon is seated inside the curve of the proximal caudal vertebrae of a skeleton planed flat by wind-blown sand at a site in Gadoufaoua, Ténéré Desert, Niger (photo by M. Hettwer). (0.34 MB TIF) [file pone.0001230.s001.tif]

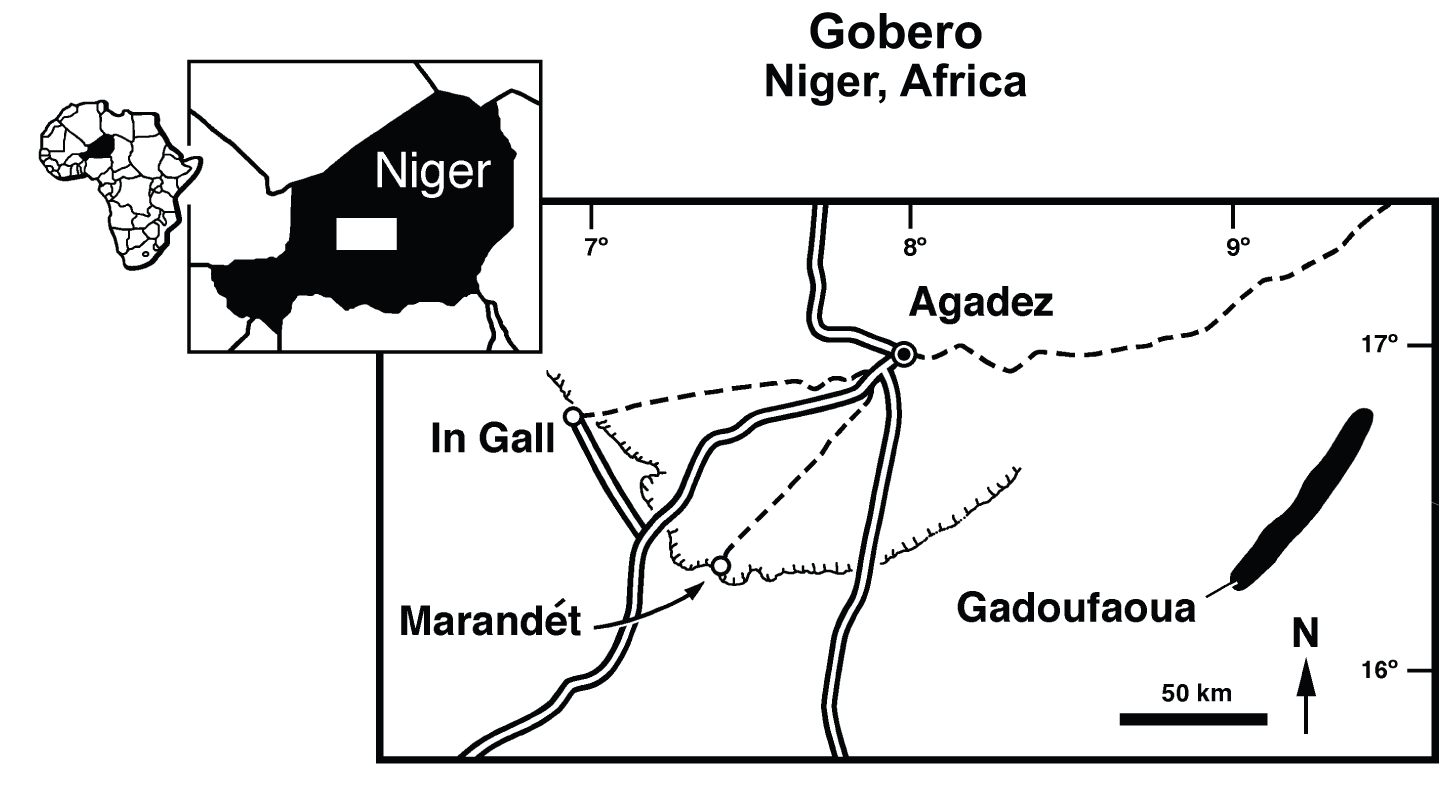

Supplement: Figure S2 — Location of outcrops of the Elrhaz Formation where fossils of Nigersaurus taqueti were found. (0.13 MB TIF) [file pone.0001230.s002.tif]

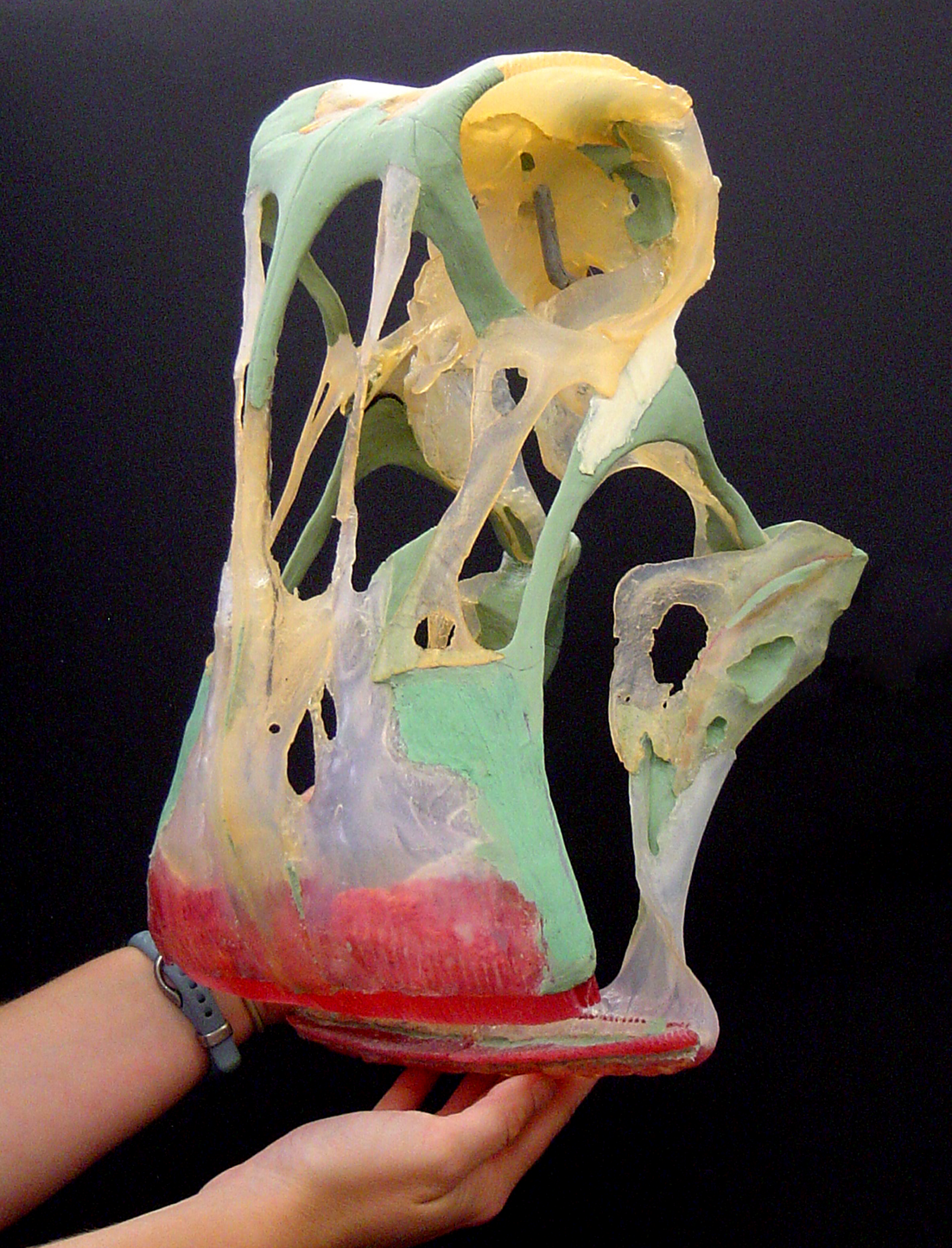

Supplement: Figure S3 — Assembled semi-translucent skull model of Nigersaurus taqueti built from prototyped skull bones (tooth batteries and reconstructed teeth in red) with unknown bones in green modeling clay (photo by T. Keillor). (3.47 MB TIF) [file pone.0001230.s003.tif]

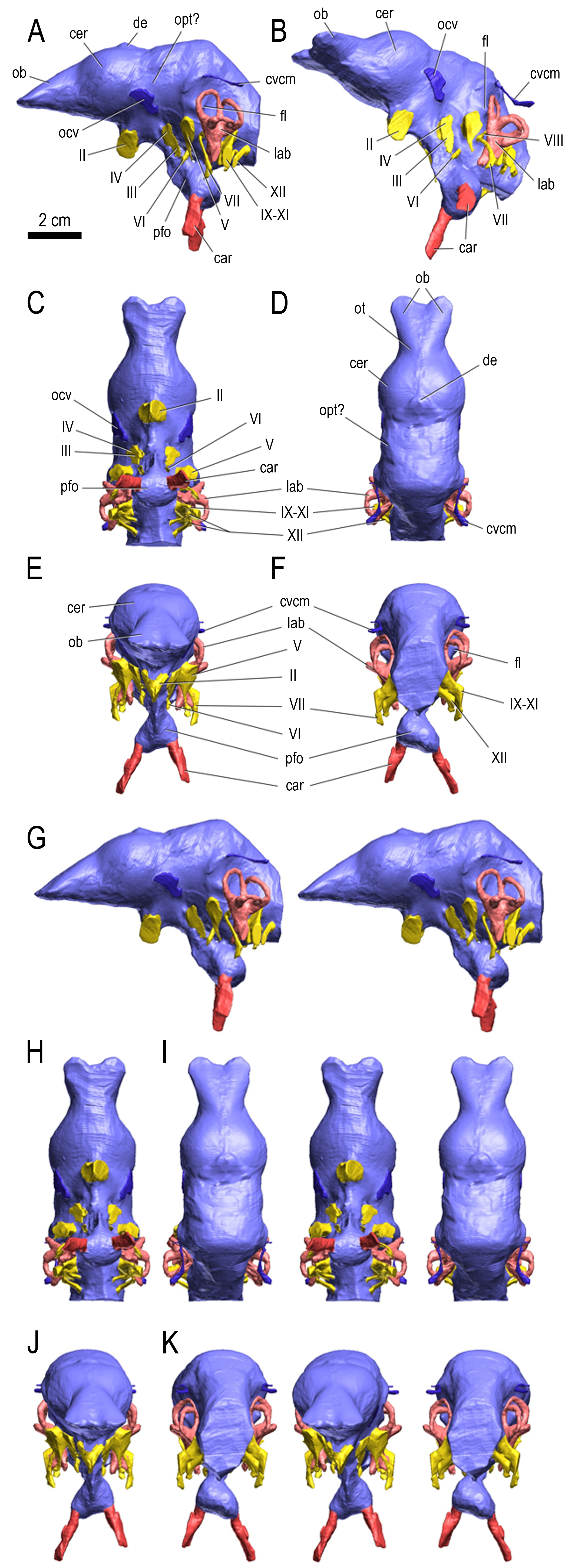

Supplement: Figure S4 — Cranial endocast, endosseous labyrinth, and some endocranial vascular structures in Nigersaurus taqueti (MNN GAD512) derived from surface renderings of µCT scan data. (A)-left lateral view. (B)-left anteroventrolateral view. (C, H)-ventral view. (D, I)-dorsal view. (E, J)-anterior view. (F, K)-posterior view. Color scheme: cyan blue, cranial endocast; pink, endosseous labyrinth; yellow, nerve canals (some of which also transmit veins); red, arterial canals; dark blue, smaller venous canals. Abbreviations: car, cerebral carotid artery canal; cer, cerebral hemisphere; cvcm, caudal middle cerebral vein; de, dural expansion; fl, flocculus ( = cerebellar auricle); lab, endosseous labyrinth; ob, olfactory bulb; ocv, orbitocerebral vein; opt?, possible optic tectum ( = lobe); pfo, pituitary ( = hypophyseal) fossa; II, optic nerve canal; III, oculomotor nerve canal; IV, trochlear nerve canal; V, trigeminal nerve canal; VI, abducens nerve canal; VII, facial nerve canal; VIII, canal for vestibular branch of vestibulocochlear nerve; IX-XI, shared canal for glossopharyngeal, vagus, and accessory nerves and accompanying vessels; XII, hypoglossal canal. (7.88 MB TIF) [file pone.0001230.s004.tif]

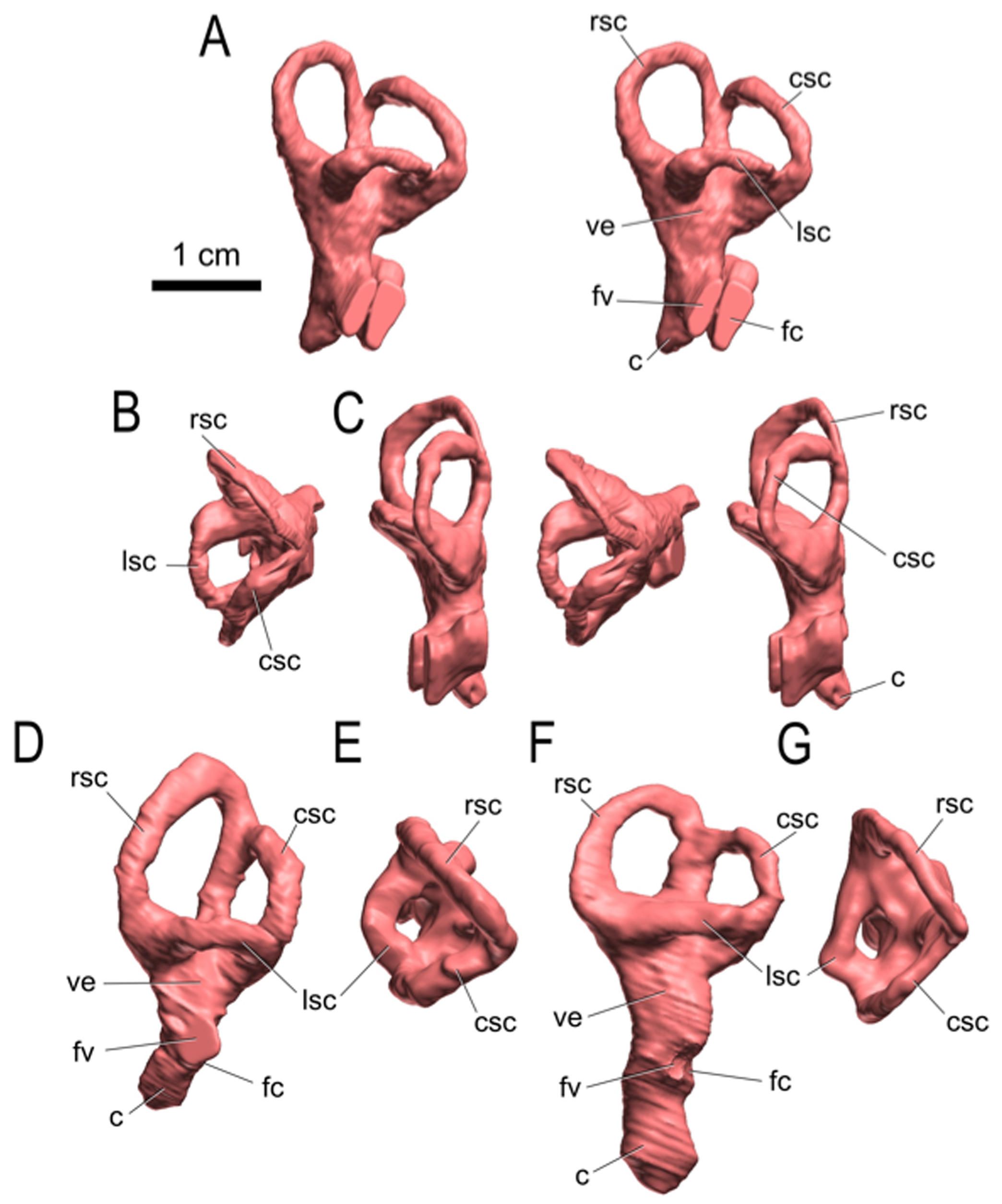

Supplement: Figure S5 — Endosseous labyrinths of the left inner ear of (A-C, stereopairs) the rebbachisaurid Nigersaurus taqueti (MNN GAD512), (D-E) the diplodocid Diplodocus longus (CM 11161), and (F-G) the basal neosauropod Camarasaurus lentus (CM 11338). (A, D, F)-left lateral view. (B, F, G)-dorsal view. (C)-posterior view. Abbreviations: c, cochlea; csc, caudal (posterior vertical) semicircular canal; fc, fenestra cochlea ( = round window); fv, fenestra vestibuli ( = oval window); lsc, lateral (horizontal) semicircular canal; rsc, rostral (anterior vertical) semicircular canal; ve, vestibule of inner ear. (2.41 MB TIF) [file pone.0001230.s005.tif]

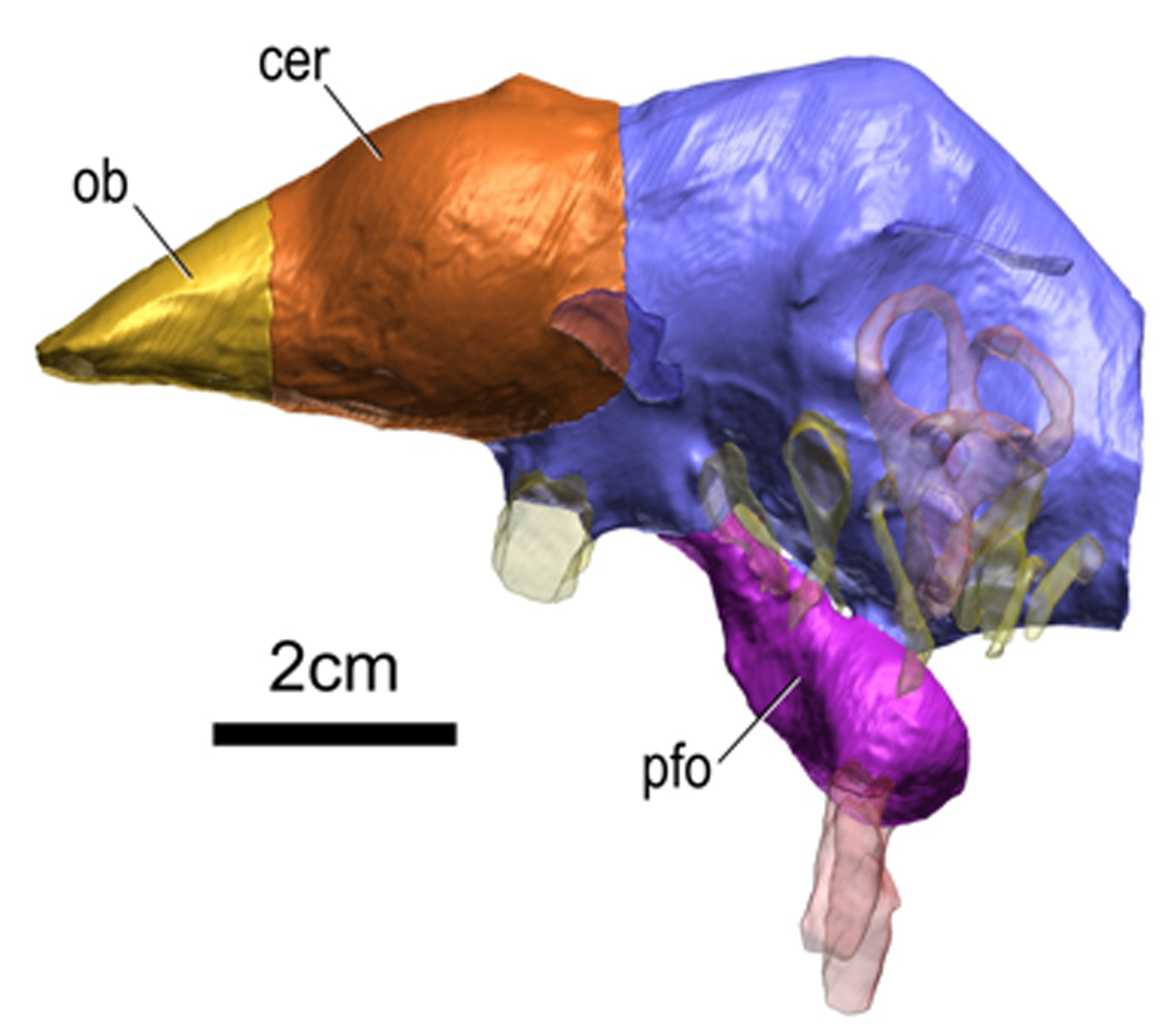

Supplement: Figure S6 — Partitioned endocast with transparent osseous labyrinth from Nigersaurus taqueti. Colors highlight the partitions used for digital assessment of endocast volumes. (1.27 MB TIF) [file pone.0001230.s006.tif]
