# Supplementary material for: Structural Extremes in a Cretaceous Dinosaur
Source: PLoS One. 2007 Nov 21;2(11):e1230. doi: 10.1371/journal.pone.0001230 (PMC2077925; doi:10.1371/journal.pone.0001230)
Supplement: Text S1 — Geologic setting. (0.07 MB PDF) [file pone.0001230.s007.pdf]

TEXT S1: GEOLOGIC SETTING

El Rhaz Formation

The bones of *Nigersaurus taqueti* were found in the El Rhaz Formation of the Tegama Group, which consists of a thick sequence of coarse-to-medium grained, cross-bedded sandstones almost devoid of finer-grained horizons [1,2] (Figure 1S). The beds are considered to be Aptian-to-Albian in age, and the localities in the Ténéré Desert (dubbed "Gadoufaoua") were a considerable distance from deltaic habitats in the region of the Benue Trough (present day Nigeria) (Figure 2S). The aquatic fauna recovered is entirely freshwater.

Nigersaurus was one of the most common large herbivores of its day, judging from the number of specimens collected. Its bones are exceeded in number only by the more robust iguanodontian *Lurdusaurus* [3]. Together, these two herbivores shared their riparian habitat with two relatives, an unnamed titanosaur [2] and the iguanodontian *Ouranosaurus* [4], composing one of the few megaherbivore associations (herbivores more than 10^6 g) with a balance of sauropods and large ornithopods. Predators would have included the giant crocodylomorph S*arcosuchus imperator* [5], the spinosaurid *Suchomimus tenerensis* [6], and similar sized basal abelisaurid and carcharodontosaurid theropods [7].

REFERENCES

1. Taquet P, Russell DA (1999) A massively-constructed iguanodont from Gadoufaoua, Lower Cretaceous of Niger. Ann Paleontol 85: 85-96.
2. Sereno PC, Beck AL, Dutheil DB, Larsson HC, Lyon GH et al. (1999) Cretaceous

sauropods from the Sahara and the uneven rate of skeletal evolution among dinosaurs. Science 286: 1342-1347.

3. Taquet P, Russell DA (1999) A massively-constructed iguanodont from Gadoufaoua, Lower Cretaceous of Niger. Ann Paleontol 85: 85-96.
4. Taquet P (1976) Géologie et paléontologie du gisement de Gadoufaoua (Aptian du Niger). Cah Paléontol 1976: 1-191.
5. Sereno PC, Larsson HCE, Sidor CA, Gado B (2001) The giant crocodyliform *Sarcosuchus* from the Cretaceous of Africa. Science 294: 1516-1519.
6. Sereno PC, Beck AL, Dutheil DB, Gado B, Larsson HCE et al. (1998) A long-snouted predatory dinosaur from Africa and the evolution of spinosaurids. Science 282: 1298-1302.
7. Sereno PC, Brusatte SL (in press) Basal abelisaurid and carcharodontosaurid theropods from the Elrhaz Formation (Aptian-Albian) of Niger. Acta Palaeontol Pol.
