# Supplementary material for: Structural Extremes in a Cretaceous Dinosaur
Source: PLoS One. 2007 Nov 21;2(11):e1230. doi: 10.1371/journal.pone.0001230 (PMC2077925; doi:10.1371/journal.pone.0001230)
Supplement: Text S2 — The prototype skull. (0.08 MB PDF) [file pone.0001230.s008.pdf]

TEXT S2: THE PROTOTYPE SKULL

***µ*CT Scanning**

High-resolution *µ*CT scans of all cranial bones and of the skull cast were completed at the High-Resolution X-ray Computed Tomography Facility at the University of Texas at Austin (UTCT). Scans were made for two principal reasons:

(1) To see internal and cross-sectional morphologic information

(2) To replicate and/or reverse the very delicate bones without damage

A portion of the stapes including the footplate is present in place in the braincase. The isolated but closely associated skull and neck bones of MNN GAD512 (MNN = Musée National du Niger) are preserved with little or no distortion. Once freed of the sandstone matrix, the bones are exceptionally fragile, given their lightweight construction. Many of the cranial bones, for example, are thin enough a strong beam of light will through them.

Assembly of the Prototype Skull

Stereolithographic (STL) prototypes at the highest resolution were made from the scans of the bones by Laser Modeling, Inc. (Schaumberg, Illinois). STL employs an ultraviolet laser to selectively cure a liquid plastic resin. This process produces a very detailed part and allows reflecting these parts to create any missing contralateral bones. Many of the bones of the skull were preserved on one or the other side, and the braincase was preserved past the midline. Thus, we recreated the missing portion of the braincase via reflection and any missing opposing elements by reflecting the parts that were preserved.

The following bones of the dorsal skull roof and braincase are not known and were filled in with modeling putty: nasal, lacrimal, quadratojugal, prefrontal, parietal, and supraoccipital. The lateralmost portion of the maxillary also is not known. For the palate, both quadrates are preserved in the holotype. An isolated palatine from a smaller individual is also preserved. The pterygoid and vomer are not known. In the lower jaw, the three main external bones are all that are preserved (dentary, surangular, angular).

To our knowledge, this is the first dinosaur skull rebuilt from STL prototypes of its constituent bones (Figure 3S). These nearly distortion-free bones and their reflected casts (when needed) allowed very little latitude in skull shape during reconstruction. The premaxilla and maxilla articulate and establish the width of the upper jaws and the anterior part of the internasal bar. The well preserved, slender ascending process of the maxilla is complete, and leaves little doubt as to where the joint with the missing lacrimal and nasal would have been. The frontal, postorbital, squamosal, braincase and quadrate articulate and match the muzzle unit when brought together. The only bone of these that articulates with any freedom of movement is the quadrate (because of the missing pterygoid and quadratojugal). Its position, however, is well established by the location of the jaw joint, which can be determined on the surangular (i.e. an everted, thickened edge). The relation of the muzzle and braincase units, thus is well established, as is the position of the occipital condyle, which is positioned ventral to the braincase between and nearly level with the heads of the right and left quadrate.

The bony struts connecting muzzle and braincase units are remarkably weak. The premaxilla, maxilla and jugal never exceed 2 mm in thickness. Their minimum

widths between these two skull units are 8, 5, and 10 mm, respectively, for a total cross-sectional area of 46 mm^2. Missing bones that would have also bridged these units include the quadratojugal, palatine and vomer. The palatine is known from a smaller individual and is a thin, plate-shaped bone as in *Diplodocus*. Assuming the quadratojugal and vomer would be comparably thin (judging from other sauropods), there may be as much as 50 mm^2 of bridging bone missing. The total bone cross-sectional area, thus, is likely to have been as little as 1.0 cm^2. This is all the more remarkable, given that the functioning tooth row is located at the distal end of the muzzle unit.
