# Supplementary material for: Structural Extremes in a Cretaceous Dinosaur
Source: PLoS One. 2007 Nov 21;2(11):e1230. doi: 10.1371/journal.pone.0001230 (PMC2077925; doi:10.1371/journal.pone.0001230)
Supplement: Text S3 — Endocast and labyrinth. (0.15 MB PDF) [file pone.0001230.s009.pdf]

TEXT S3: ENDOCAST AND LABYRINTH

Methods

The original fossil elements were CT scanned at the High-Resolution X-ray Computed Tomography Facility at the University of Texas at Austin (UTCT). A physical endocast (using traditional latex techniques) was prepared in the Fossil Laboratory at the University of Chicago from an assembled braincase compose of stereolithographic replicas of the preserved elements, mirrored elements (e.g. left frontal), and one restored element (parietal). A resin replica of the physical endocast was subsequently scanned (slice thickness of 92 µm) at the Ohio University MicroCT facility (OUµCT).

A virtual endocast and endosseous labyrinth of the inner ear was created by combining the scan data of the braincase from both UTCT and OUµCT (Figures 4S, 5S). Structures of interest were highlighted (i.e. segmented) from the UTCT scan data of the original fossils using Amira v. 4.1.2. The OUµCT scan data of the physical endocast were registered to the virtual endocast in Amira, allowing addition of the restored parietal-supraoccipital region. Finally, the missing parts of the right side of the endocast, as well as the missing right labyrinth, were added in Amira by mirroring the preserved portions of the left side. Both surfaces and volumes were generated and used to generate Figure 4S and those in the paper (Figure 1F, G). To facilitate discussion, we will refer to the digital casts of structures as if they were the structures themselves (e.g. 'olfactory bulb'). We present additional information on the endocast and labyrinth below; comparative data on the sauropods *Camarasaurus* and *Diplodocus* are published elsewhere [1].

Cranial Endocast

Unlike other sauropods in which an endocast has been prepared, *Nigersaurus* had relatively small dural sinuses over the cerebrum. For the first time among sauropods, many brain divisions and features are discernible including the cerebrum, floccular lobe of the cerebellum, and possibly the optic tectum (optic lobes).

The cerebral hemispheres of *Nigersaurus* are not particularly expanded relative to other sauropods, although this comparison is difficult to make as the cerebrum is not fully exposed in other sauropods. The best comparative measure is the lateral extent of the cerebrum relative to the lateral extent of the endosseous labyrinth in dorsdal view (Figure S4). In *Camarasaurus* and *Diplodocus*, the cerebral portion of the endocast extends laterally about as far as the lateral margin of the lateral semicircular canal [1]; the cerebrum of *Nigersaurus* is comparable or little narrower, probably due smaller venous sinuses.

The small olfactory bulbs lie in front of the cerebrum and are connected by very short olfactory tracts (Figure S4). As in other sauropods, they diverge anteriorly. Compared to other sauropods, the bulbs are small and the tracts almost nonexistent. In other sauropods, the olfactory bulbs also angle strongly dorsally. The marked angulation of the olfactory tracts and bulbs in other sauropods is probably due to the strong retraction of the olfactory region of the nasal cavity (between the orbits in most sauropods). The absence of such angulation in *Nigersaurus* is a byproduct of extreme downward rotation of the muzzle, and a compensatory straightening of the olfactory apparatus.

Rounded swellings behind the cerebral hemispheres on each side may represent the optic tecta or alternatively a foramen for the orbitocranial vein (Figure S4A, B, D). A small floccular lobe of the cerebellum is clearly discernible, the first recorded in a sauropod endocast. The flocculus (cerebellar auricle) in extant birds is involved in the vestibulo-ocular reflex. The cranial nerve foramina, pituitary fossa, cerebral carotid canal are fairly typical of sauropods, although the foramina opening into the orbit are somewhat enlarged.

Endosseous Labyrinth

The left side of MNN GAD512 preserves a complete endosseous labyrinth (Figure S5). It is similar to other sauropods in having an enlarged vestibular region above the fenestra vestibuli and adjacent to the confluence of the semicircular canals. The cochlea (lagena) is somewhat shorter than in other sauropods [1], suggesting that discrimination of air-borne sounds may have been less important in *Nigersaurus*. The semicircular canals are sensitive to acceleration and have important neural links to the eye muscles. Animals with well developed gaze stabilization mechanisms tend to have elongate canals and also tend to be relatively agile, mobile, and jerky in their movements [2]. Sauropods, not surprisingly, have relative short and thick semicircular canals, reflecting diminished gaze stabilization mechanisms [1]. *Nigersaurus*, however, has relatively elongate canals for a sauropod, particularly the lateral canal that may have enhanced control of lateral scanning movements of the head. The differentiation in the endocast of flocculi and optic tecta as well suggests that *Nigersaurus* may have had somewhat enhanced gaze stabilization mechanisms over some other sauropods.

Body and Brain Mass

Body mass of approximately 4 metric tons was estimated by reducing the body mass body calculated for the similarly proportioned, slightly larger (12-meter long) diplodocoid *Dicraeosaurus*. Two mass estimates of 4,421 and 5,400 kg were given recently for this sauropod using the alternative methods of Seebacher [3] and Christiansen [4], respectively. A body mass estimate for *Amargasaurus* of 6853 kg [3] seems high for a sauropod with an estimated length of 10 m, or less than that of *Dicraeosaurus*.

Endocast volumetrics derived from the braincase of the holotypic specimen (MNN GAD512; MNN = Musée National du Niger). Erosion removed about one-third of the left side. The braincase was scanned, prototyped, and reverse-prototyped. The reverse copy was cut to complete the braincase, with the exception of the missing parietal and supraoccipital; the skull roof in this region was finished to fit all sides. *Nigersaurus* has a reduced dorsal dural sinus much smaller than that in *Camarasaurus* and *Diplodocus* (Figure 1G). The only indication of this sinus in *Nigersaurus* is a shallow median depression toward the distal end of the frontal (MS-Figure 1G). As a result, the cerebrum is fully exposed in *Nigersaurus*, and the volume for this portion of the endocast can be measured accurately for the first time in a sauropod.

A standard physical endocast was prepared from the skull roof and ventral portion of the braincase and cut apart (by PCS) to measure volumes by water displacement in a graduated cylinder. The endocast was trimmed fore and aft following Osborn [5], using the constriction between cerebrum and olfactory bulbs (olfactory tracts) anteriorly and immediately posterior to the exits for CN XII posteriorly. This

volume was divided into forebrain (cerebrum) and remainder of the endocast, with nerve stalks trimmed flush. A complete bulb was cut by extending the marked depressions into an ovoid. Water displacement gave volumes of 4, 15, 27 and 42 cm^3, for paired olfactory bulbs, forebrain (cerebrum), mid and hindbrain, and total endocast volume (fore, mid and hind brain), respectively. Independently, these volumes were estimated digitally from a μCT scan of the braincase (by LMW), which also provided the chance to measure the volume of the pituitary fossa (Figure 6S). These very comparable volumetric estimates are the ones we used in the paper (2.9, 16.6, 33.9 and 53.4 cm^3, for paired olfactory bulbs, forebrain, mid/hindbrain, and total endocast volume).

We assessed brain versus body mass in *Nigersaurus* using 95% confidence intervals from a log-log regression based on nonavian reptiles [6]. Although noticeably smaller in volume than comparably sized theropods, we found its brain mass to plot within the 95% confidence limits, using the mass estimate discussed above.

Another popular measure is the Encephalization Quotient (EQ), which presents a comparison of actual brain mass relative to the expected brain mass for an animal of its body size. There are some problems with current methods, because the formulae were not generated with currently acceptable comparative methods. EQ, nonetheless, may constitute a reasonable first approximation. To this end, we used Hurlburt's [7] equation based on extant nonavian reptiles that yields an REQ (Reptile EQ): REQ = MassBrain/0.0155 x MassBody0.553. We calculate MassBrain by multiplying endocast volume by the density of brain tissue (1.036 g cm-3). Typically, a correction factor of 50% is used to account for the mismatch (i.e. MassBrain equals 50% of endocast mass). In *Nigersaurus* the reduced dural venous sinuses may shift the proportion

above 50%. Thus we calculated REQ values using the '50% rule' and a '100% rule' (brain and endocast mass are equal). Using an endocast volume of 53.4 cm3, a 100% MassBrain of 55.32 g, a 50% MassBrain of 27.66 g, and a body mass of 4 x 106 g, REQ50% rule equals 0.40 and REQ100% rule equals 0.80.

These values suggest that *Nigersaurus* had a relatively small brain 40–80% the size expected for a reptile of its body size. Witmer et al. [1] calculated the REQ for *Diplodocus* with its larger sinuses using the '50% rule' and arrived at a value of 0.41, almost identical to that for *Nigersaurus*. Pending further study, we conclude that *Nigersaurus* and other sauropods do have relatively smaller brains that other dinosaurs and perhaps rank in the lower range of brain mass among extant nonavian reptiles.

REFERENCES

1. Witmer LM, Ridgely RC, Dufeau DL, Semones MC (in press) Using CT to peer into the past: 3D visualization of the brain and ear regions of birds, crocodiles, and nonavian dinosaurs. In: Frey R, Endo H, eds. Anatomical Imaging: Towards a New Morphology. Tokyo: Springer-Verlag.
2. Spoor F, Garland ST, Krovitz G, RyanTM, Silcox MT, and Walker A (2007) The primate semicircular canal system and locomotion. PNAS 104:10808–10812.
3. Seebacher F (2001) A new method to calculate allometric length-mass relationships of dinosaurs. J Vert Paleont 21: 51-60.
4. Christiansen P (1997) Locomotion in sauropod dinosaurs. GAIA 14: 45-75.
5. Osborn HF (1912) Crania of *Tyrannosaurus* and *Allosaurus*. Mem Amer Mus Nat Hist 1: 1-30.

6. Larsson HCE, Sereno PC, Wilson JA (2000) Forebrain enlargement among nonavian theropod dinosaurs. J Vert Paleont 20: 615-618.
7. Hurlburt, GR (1996) Relative brain size in Recent and fossil amniotes: determination and interpretation. PhD Dissertation, University of Toronto, Toronto, Ontario, Canada, 250 pp.
