# Supplementary material for: Structural Extremes in a Cretaceous Dinosaur
Source: PLoS One. 2007 Nov 21;2(11):e1230. doi: 10.1371/journal.pone.0001230 (PMC2077925; doi:10.1371/journal.pone.0001230)
Supplement: Text S4 — Microwear and incremental lines of von Ebner. (0.13 MB PDF) [file pone.0001230.s010.pdf]

TEXT S4: MICROWEAR AND INCREMENTAL LINES OF VON EBNER

Methods for Assessing Wear

Eight teeth referred to *Nigersaurus taqueti* were examined for microwear features on enamel and dentine surfaces using a light microscopy-based method [1]. Surfaces were cleaned before molding with 70% isopropyl alcohol to remove surface dust or dirt. Two molds were made for the worn surface of each tooth using a polyvinylsiloxane mold material (President Microsystem 6012, Coltene). The first cast was discarded as any surface artifacts or debris would adhere. High-quality clear epoxy resin was used to make the second cast of each tooth. Wear facets were examined under a light microscope at 70x magnification using reflected light. Wear features were examined in a 0.3mm x 0.3mm area and sorted into four categories: fine scratches (narrow and shallow), coarse scratches (wider and deeper), pits roughly circular depressions), and gouges (irregularly shaped or oblong pits). Preservation permitting, up to four sites were examined on each facet to survey areas of enamel and dentine to eliminate variation. Images were taken using a Spot CCD camera (Spot Insight 11.2 Color Mosaic, Diagnostic Instruments) at the highest resolution available (36 bits/pixel), mounted on a Nikon SMZ 1500 microscope.

Description

Nigersaurus taqueti is characterized by teeth with paired wear facets, located opposite each other on the external and internal surfaces of each crown (Figure 2), with angles of about 40° and 5° to the crown axis (as measured from the straighter distal portion of the crown near the facets). The external, or labial, facet is the smaller of the two, and cuts

the tooth at a comparative shallow angle. This facet is notable for the presence of a deep trough formed at the enamel-dentine junction, a feature commonly produced by abrasion during tooth-food contact. The concavity is deepest at the base of the facet, indicating tooth abrasion occurred in a predominantly crown-root direction, although in several teeth this facet is slightly asymmetrical, with the apex slightly offset from the midline of the tooth. High-angled facets of this type are also known from the other two main radiations of diplodocoid sauropods, Diplodocidae (*Diplodocus,* CM 1161; CM = Carnegie Museum) and Dicraeosauridae (*Dicraeosaurus,* MB.R. 2197, 2204; MB = Humbolt Museum).

The second facet, located lingually, is proportionally much longer and occurs at a much steeper angle relative to the long axis of the tooth. Unlike the external facet's rounded, U-shaped outline, the internal facet is an elongate trapezoid with a very narrow base. The margins of this facet are smooth and rounded, indicative of attrition caused by tooth-tooth contact, not abrasive contact with plant material or grit. Kellner (1996) figured a tooth (DGM 907-R; DGM = Departamento Nacional de Produçao Mineral) from the Late Cretaceous Bauru Group of southeastern Brazil, which also has a similar pair of labial and lingual wear facets. Although Kellner tentatively considered the tooth to belong to a titanosaur on the basis of the abundance of titanosarian bones from the Bauru Basin, their similarity to *Nigersaurus* is strking [2].

Eight isolated teeth referred to *Nigersaurus* were examined for dental microwear using the light microscopy method of Solounias and Semprebon (2002). All are probably upper teeth, as all have high-angle wear facets on their internal surfaces; lower teeth may have been destroyed in the course of wear [2]. Both lingual and labial facets were

examined for the presence of fine and coarse scratches, pits (roughly circular depressions), and gouges (irregularly shaped or oblong pits). Three teeth were found to be completely unusable due to high levels of taphonomic abrasion. Of the remaining five teeth, most showed evidence of taphonomic alteration, but in each case microwear features were still present and identifiable on at least one aspect of the tooth. Commonly, both on teeth selected for potential features and some remaining that were eliminated before examination, the thin enamel surface of the lingual aspect of the tooth had been worn away, either through use or by taphonomic processes, and no trace of microwear could be found. Those cases are characterized by a coarse, pock-marked surface, and in one case wear so severe that it exposed the pulp cavity.

Labial Facet

Microwear features are most commonly noted on the enamel surfaces of diplodocoid sauropods, but in *Nigersaurus* the labial rim often lacks plain wear features and is instead highly polished, and wear features are more obvious on the dentin. This polishing is not reminiscent of taphonomic alteration by acids, bases, or sand abrasion [3], and is more likely a result of frequent and heavy wear by finely abrasive materials. Microwear features that are present are typically limited to fine scratches oriented roughly labio-lingually, with some inclination medially or laterally, in some cases following the curve of the enamel-dentine contact. Where microwear is quantifiable, feature density is high (7-16 scratches per measurement unit), but within the range noted for *Diplodocus*. Pits are present but rare, gouges and coarse scratches are absent in the enamel surface. Wear features often extend for a short distance onto the

labial face of the tooth, basal to the facet. Some gouges are present on the enamel surface basal to the facet of one tooth. On one tooth without an apical facet, long, coarse scratches occur across the entire labial enamel surface, oriented between roughly 33º and 45º to the long axis. Microwear features were also noted on the dentine surface–these were less common (4-11 features per unit study, avg. 7.5) but typically much larger. Wear again consists primarily of fine scratches, often very long (0.1-0.2mm), with both gouges and pits present, possibly due to the softer nature of dentin.

Lingual Facet

Microwear was also noted on the lower-angle facet on the lingual face, although this surface is much more commonly taphonomically abraded beyond use for microwear study; even when features are present, they often occur in oases between heavily abraded regions. These features again consist primarily of long, fine scratches distributed equally across the facet, with pitting and gouging also common. The lateral edges of the facet are characterized by polished edges at coarser magnifications, but at 70x magnification the presence of large, deep scratches oriented along the crown-root axis occasionally appear at the lateral margins of the facet. This is accompanied by irregularly edged pits and gouges. Enamel attrition due to tooth-tooth contact is usually associated with “plucking” of prisms in prismatic mammalian enamel, but its impact on the aprismatic, columnar enamel typical of Diplodocoidea [4] has not been studied; these coarse wear features may represent the results of a similar process. The apex of this facet is typically without features, but occasionally fine scratches oriented along the crown-root direction do appear.

Microwear Summary

The pattern of microwear remaining on the labial facet, with dominance of fine scratches over pits, gouges, and deep scratches, is typical of a diet lacking hard foods (such as seeds) or high amounts of grit [5], although the deep excavation and highly polished enamel surface indicate a highly abrasive foodstuff. In particular, long scratches such as seen in the dentine have been associated with "softer" diets [6]. This agrees with the wear suggested by the presence of a deep erosional "lip" formed at enamel-dentine junction, indicative of heavy wear caused by shearing contact with plant material. Combined with asymmetrical enamel that is thickest on the external face, these data suggest that the small facet represents the "functional" side of the tooth. Dietary inferences in wholly extinct lineages are without much power beyond qualitative assessments, and so no attempt to quantify specific preferred foods of *Nigersaurus* will be made here.

Scratch orientation is variable between teeth but cross scratching is not noted. In each case, predominant scratch orientation is at a slight angle to the long axis. This would seem to indicate a predominantly unidirectional shearing motion with upper and lower teeth meeting essentially parallel to each other. Deviation from the crown-root axis is potentially a function of slightly imperfect occlusion or tooth orientation within the alveolus – the dentition of upper and lower jaws differ in the number of teeth present and the size of those teeth, which may contribute to uneven or off-center wear. The slight asymmetry in the shape of the facet may also be the result of this type of imperfect occlusion. If the upper teeth occlude off-center of the lower teeth, the lateral

offset between the points of food contact in the upper and lower teeth will determine the offset in the apex of the labial wear facet. No indication of side-to-side slicing action of the jaws is suggested by the microwear features, and propaliny seems unlikely, given the lack of an attritional facet on the labial surface of any examined teeth.

The internal facet seems to indicate wear primarily by tooth-tooth contact, although microwear features typical of tooth-food contact are also present. The predominance of attritional macrowear features and thin enamel surface suggest that the lingual aspect does not represent a functional food-processing surface, and any tooth-food contact was incidental. As on the labial aspect, the predominantly crown-root orientation of microwear features suggests a vertical slicing action, as does the essentially midline location of the facet apices. The presence of these microwear features, however, reinforces the assertion of [2] that this surface must have been exposed and not covered by a successive tooth in a tooth battery. Although it is possible that each tooth did not meet the tooth labial to it, this seems unlikely in a tooth battery. It is possible that this wear occurred only when the tooth was exposed as the lingual-most rank in the tooth battery “phalanx”, and that as the tooth advanced and successive teeth filled in behind it, this surface was no longer unprotected. Given the relative thinness of the enamel on this surface, *Nigersaurus* may have minimized exposure (and therefore wear) on the lingual aspect in this way.

Incremental Lines of von Ebner

Lines of von Ebner were assessed by longitudinal and transverse sections of an intact premaxillary tooth battery that preserved successional teeth in the first and second

columns from the midline. In cross-section of two successional crowns in the second tooth column, approximately 60 and 40 incremental lines of von Ebner are visible for a cross-sectional difference of about 20 lines (days). The age of a tooth and the rate of replacement, however, must ultimately be assessed by counting lines in longitudinal section, because the cone-shaped accumulation of dentine during crown growth [7].

REFERENCES

1. Solounias N, Semprebon G (2002) Advances in the reconstruction of ungulate ecomorphology with application to early fossil equids. Amer Mus Novitates 3366: 1-49.
2. Sereno PC, Wilson JA (2005) Structure and evolution of a sauropod tooth battery. In: Rogers KAC, Wilson JA, eds. The Sauropods: Evolution and Paleobiology. Berkeley: University of California Press. pp. 157-177.
3. King T, Andrews P, Boz B (1999) Effect of taphonomic processes on dental microwear. Amer J Phys Anthropol 108: 359-373.
4. .Sander, PM (1999) The microstructure of reptilian tooth enamel: terminology, function, and phylogeny. Münch geowiss Abh (Reihe A) 38:1-102.
5. Teaford, MF (1985) Molar microwear and diet in the genus *Cebus*. Amer J Phys Anthropol 66:363-370.
6. Goswami A, Flynn JJ, Ranivoharimanana L, Wyss AR (2005) Dental microwear in Triassic amniotes: implications for paleoecology and masticatory mechanics. J Vert Paleont 25: 320-329.

7. Erickson GM (1996) Incremental lines of von Ebner in dinosaurs and the assessment of tooth replacement rates using growth line counts. Proc Natl Acad Sci USA 93: 14623-14627.
