# Supplementary material for: Structural Extremes in a Cretaceous Dinosaur
Source: PLoS One. 2007 Nov 21;2(11):e1230. doi: 10.1371/journal.pone.0001230 (PMC2077925; doi:10.1371/journal.pone.0001230)
Supplement: Text S5 — Phylogenetic analysis. (0.16 MB PDF) [file pone.0001230.s011.pdf]

TEXT S5: PHYLOGENETIC ANALYSIS

The phylogenetic analysis yields 5 shortest trees with one polytomy among ingroups, that between the partially known diplodocoid *Suuwassea*, Dicraeosauridae, and Diplodocidae. Two of the five trees represent alternative arrangements among the outgroups *Omeisaurus*, *Jobaria* and Macronaria. Although there exist only a few shortest trees, tree number increases rapidly when considering trees even one step longer. The reason for this is the high level of missing data in several of the rebbachisaurids included in the analysis. *Rebbachisaurus*, in particular, can be scored postively for only 19 of 102 characters (19% of the data). Limiting ingroups to taxa with less than 50% missing data increases the robustness of the tree but removes most rebbachisaurids with the exception of *Nigersaurus* and *Limaysaurus*.

Character List

The following list includes 102 character statements, 40 and 62 of which code cranial and postcranial variation, respectively. Character format and structure follows recent recommendations [1]. Character statements are attributed to the author that first used them in a qualitative or quantitative cladistic analysis. Characters without attribution are new to this analysis.

Cranial

1. Subnarial foramen and anterior maxillary foramen, position: well distanced from one another (0); separated by narrow bony isthmus (1). [2]
2. Premaxillary anterior margin, shape: without step (0); with marked step, anterior portion of skull sharply demarcated (1). [2]
3. Anteroventrally orientated vascular grooves originating from an opening in the maxillary contact on the premaxilla: absent (0); present (1). [2]
4. Antorbital fenestra, maximum diameter: much shorter than maximum diameter of

orbit (0); subequal to orbital maximum diameter (1). [2]

5. Dorsal margin of antorbital fenestra, shape: straight or convex (0), concave (1). [2]
6. Dorsal process of maxilla, posterior extent: anterior to or even with posterior margin of maxilla (0); extending further posterior than body of maxilla (1). [2]
7. External nares, position: terminal (0); retracted to level of orbit (1); retracted to a position between orbits (2). [2]
8. External nares, maximum diameter: shorter than maximum orbital diameter (0); longer than orbital maximum diameter (1). [2]
9. Orbital ventral margin, anteroposterior length: broad, with subcircular orbital margin (0); reduced, with acute orbital margin (1). [2]
10. Quadratojugal, position of anterior terminus: posterior to middle of orbit (0); anterior margin of orbit or beyond (1) [modified from 3]
11. Maxilla, contact with quadratojugal: absent or small (0); broad (1). [modified from 3]
12. Jugal, contribution to antorbital fenestra: very reduced or absent (0); large, bordering approximately one-third its perimeter (1). [2]
13. Prefrontal, posterior process size: small, not projecting far posterior of frontal-nasal suture (0); elongate, approaching parietal (1). [2]
14. Prefrontal, posterior process shape: flat (0); hooked (1). [2]
15. Postorbital, posterior process: present (0); absent (1). [2]
16. Frontals, midline contact (symphysis): sutured (0); fused in adult individuals (1). [2]
17. Frontal, anteroposterior length: approximately twice transverse breadth (0); less than transverse breadth (1). [2]
18. Parietal, contribution to post-temporal fenestra: present (0); absent (1) [2]
19. Postparietal foramen: absent (0); present (1). [2]
20. Parietal, distance separating supratemporal fenestrae: shorter than long axis of supratemporal fenestra (0); twice the length of the long axis of the supratemporal fenestra (1). [2]
21. Supratemporal fenestra: present (0); absent (1). [2]
22. Squamosal, contact with quadratojugal: present (0); absent (1). [2]
23. Quadrate fossa, depth: shallow (0); deeply invaginated (1). [2]
24. Pterygoid, transverse flange (ectopterygoid process), position: posterior of orbit (0); between orbit and antorbital fenestra (1); anterior to antorbital fenestra (2). [2]
25. Supraoccipital, height: twice height of foramen magnum (0); subequal to or less than height of foramen magnum (1). [2]
26. Crista prootica, size: rudimentary (0); expanded laterally into dorsolateral process (1). [2]
27. Basipterygoid processes, length: short, approximately twice basal diameter (0); elongate, at least four times basal diameter (1). [2]
28. Basipterygoid processes, angle of divergence: approximately 45º (0); less than 30º (1). [2]
29. Basal tubera, fusion: unfused (0); fused (1). [4]
30. Basioccipital depression between occipital condyle and basal tubera: absent (0); present (1). [2]
31. Basipterygoid processes, orientation: perpendicular to skull roof (0); angled

approximately 45º to skull roof (1). [2]
32. Dentary, anteroventral margin shape: gently rounded (0); sharply projecting triangular process or 'chin' (1). [2]
33. Tooth rows, shape of anterior portions: narrowly arched, anterior portion of tooth rows V-shaped (0); broadly arched, anterior portion of tooth rows U-shaped (1); rectangular, tooth-bearing portion of jaw perpendicular to jaw rami (2). [2]
34. Tooth rows, length: extending to orbit (0); restricted anterior to orbit (1); restricted anterior to subnarial foramen (2). [2]
35. Crown-to-crown occlusion: absent (0); present (1). [2]
36. Occlusal pattern: V-shaped wear facets (0); single planar facet (1); paired planar facets (2). [modified from 2]
37. Tooth crowns, orientation: aligned along jaw axis, crowns do not overlap (0); aligned slightly anterolingually, tooth crowns overlap (1). [2]
38. Tooth crowns, cross-sectional shape at midcrown: elliptical (0); D-shaped (1); cylindrical (2) [2]
39. Dentary teeth, number: greater than 20 (0); 17 or fewer (1). [2]
40. Replacement teeth per alveolus, number: two or fewer (0); three or more (1). [modified from 2]

Postcranial

41. Dorsal pneumatopores (pleurocoels): absent (0); present (1). [2]
42. Dorsal vertebrae, height of neural arch: less than height of centrum (0); subequal or greater than height of centrum (1).
43. Dorsal vertebrae, spinodiapophyseal webbing: laminae follow curvature of neural spine in anterior view (0); laminae "festooned" from spine, dorsal margin does not closely follow shape of neural spine and diapophysis (1).
44. Atlantal intercentrum, occipital facet shape: rectangular in lateral view, length of dorsal aspect subequal to that of ventral aspect (0); expanded anteroventrally in lateral view, anteroposterior length of dorsal aspect shorter than that of ventral aspect (1). [2]
45. Cervical vertebrae, number: fewer than 10 (0); 10 (1); 12 (2); 13 (3); 14 or more (4). [2]
46. Anterior cervical neural spines, shape: single (0); bifid (1). [2]
47. Anterior cervical vertebrae, height:length ratio: height less than or roughly equal to length of centrum(0); height 1.5 times length of centrum (1). [modified from Casanovas et al. 2001]
48. Mid-cervical centra, anteroposterior length:height ratio of posterior face: 2.5-3 (0); 4+ (1). [2]
49. Middle and posterior cervical neural arches, centroprezygapophyseal lamina (cprl), shape: single (0); divided (1). [2]
50. Posterior cervical vertebrae, accessory lateral lamina connecting postzygodiapophyseal and spinoprezygapophyseal laminae: absent (0) present (1) [5]
51. Cervical vertebrae, epipophyseal-prezygapophyseal lamina (eprl): absent (0); present (1).
52. Cervical vertebrae, longitudinal ridge on ventral surface: absent (0), present (1).
53. Posterior cervical neural spines, shape: single (0); bifid (1). [modified from 2]

54. Anterior cervical vertebrae, parapophyses: without pneumatization (0); with pneumatic cavity (1).
55. Middle and posterior cervical vertebrae, parapophyses: without pneumatization (0); with pneumatic cavity (1).
56. Middle cervical vertebrae, angle between postzygodiapophyseal and spinopostzygapophyseal laminae: acute (0); approximately 90º (1). [3]
57. Mid-cervical neural spines, orientation: vertical (0); anteriorly inclined (1). [3]
58. Mid-cervical neural spines, height: approximately as high as neural arch (0); considerably higher than neural arch (1). [modified from 3]
59. Posterior cervical neural arches, accessory spinal lamina: absent (0); present, running vertically just posterior to spinoprezygapophyseal lamina (1). [2]
60. Posterior cervical neural and/or anteriormost dorsal neural spines, orientation: vertical (0); inclined anteriorly (1). [3]
61. Posterior cervical and anterior dorsal bifid neural spines, median tubercle: absent (0); present (1). [2]
62. Posterior cervical and anterior dorsal bifid neural spines, shape: widely diverging (0); narrow, parallel to converging (1). [3]
63. Dorsal vertebrae, number: 13 (0); 12 (1); 11 (2); 10 or less (3). [3, modified from 2]
64. Middle and posterior dorsal vertebrae, pleurocoels: absent (0); present (1). [modified from 3]
65. Dorsal neural spines, length: twice centrum length (0); approximately four times centrum length (1). [2]
66. Dorsal transverse processes, orientation: horizontal or only slightly inclined dorsally (0); more than 30º inclined dorsally from the horizontal (1). [3]
67. Middle and posterior dorsal neural arches, centropostzygapophyseal lamina (cpol), shape: single (0); divided (1). [2]
68. Middle and posterior dorsal neural arches, prezygoparapophyseal lamina (prpl): absent (0); present (1). [2]
69. Middle and posterior dorsal neural arches, posterior centroparapophyseal lamina (pcpl): absent (0); present (1). [2]
70. Anterior caudal neural spines, shape: tapering or not flaring distally (0); flared distally, with pendant triangular lateral processes (1). [2]
71. Posterior dorsal neural arches, hyposphene-hypantrum articulations: present (0); absent (1). [2]
72. Posterior dorsal neural spines, shape: rectangular through most of length (0); 'petal' shaped, expanding transversely through 75% of its length and then tapering (1). [2]
73. Sacral neural spines, length: twice centrum length (0); nearly four times centrum length (1). [modified from 2]
74. First caudal centrum, articular face shape: flat (0); procoelous (1); opisthocoelous (2); biconvex (3). [2]
75. Caudal neural spines, elliptical depression between spinodiapophyseal lamina and postspinal lamina on lateral neural spine: absent (0); present (1).
76. Caudal neural spines, triangular lateral processes: absent (0); present (1).
77. Anterior caudal centra (excluding the first), articular face shape: amphiplatyan (0) procoelous (1) [modified from 2]

78. Anterior caudal neural spines, spinoprezygapophyseal lamina-spinopostzygapophyseal lamina contact: absent (0); present (1). [2]
79. Anterior caudal vertebrae, lateral spinopostzygapophyseal lamina: absent (0); present (1). [6]
80. Anterior caudal transverse processes, shape: triangular, tapering distally (0); winglike (1). [2]
81. Anterior caudal transverse processes, diapophyseal laminae (acdl, pcdl, prdl, podl): absent (0); present (1). [2]
82. Anterior caudal transverse processes, anterior centrodiapophyseal lamina (acdl), shape: single (0); divided (1). [2]
83. Distalmost caudal centra, articular face shape: platycoelous (0); biconvex (1). [2]
84. Distalmost caudal centra, length-to-height ratio: less than 4 (0); greater than 5 (1). [2]
85. Distalmost biconvex caudal centra, number: 10 or fewer (0); more than 30 (1). [2]
86. Cervical ribs, length: long, overlapping several centra posterior (0); shorter than or roughly equivalent to centrum length (1). [2]
87. Chevrons, "crus" bridging haemal canal: present (0); absent (1). [2]
88. Scapula, acromion process dorsal margin: convex or straight (0); with U-shaped concavity (1). [7]
89. Scapulocoracoid, angle of articulation relative to scapular blade: 90 degrees (0); less than 90 degrees (1). [modified from 8]
90. Scapular blade, shape: acromial edge not expanded (0); rounded expansion on acromial side (1); racquet-shaped (2). [2]
91. Scapular glenoid, orientation: relatively flat or laterally facing (0); strongly beveled medially (1). [2]
92. Coracoid, anterodorsal corner shape: subrectangular (0); rounded or curved (1).
93. Humerus, midshaft cross-section, shape: circular, major and minor axes subequal (0); elliptical, major axis twice minor axis (1). [2]
94. Humerus, pronounced proximolateral corner: absent (0); present (1) [2]
95. Pubis, ambiens process development: small (0); prominent, projecting anteriorly from anterior margin of pubis (1). [2]
96. Ischial distal shaft, shape: bladelike, medial and lateral depths subequal (0); triangular, depth of ischial shaft increases medially (1). [reversed from 2]
97. Ischial distal shafts, cross-sectional shape: V- shaped, forming an angle of nearly 50° with each other (0); flat, nearly coplanar (1). [2]
98. Ischium, iliac peduncle shape: straight or widening in smooth curve distally (0); narrow, with distinct "neck" (1).
99. Ischium, elongate muscle scar on proximal end: absent (0); present (1).
100. Distal end of ischial shaft expanded: absent (0); present (1).
101. Femur, pronounced ridge on posterior surface between greater trochanter and head: absent (0); present (1).
102. Metatarsal I distal condyle, posterolateral projection: absent (0); present (1). [2]

Character–Taxon Matrix

The following are scores for 102 characters in 13 terminal taxa and 3 outgroups. Characters include state “0” (= primitive), states “1-4” (derived), “?” for missing information, and “9” for inapplicable data. All characters are binary except 11 (characters 8, 26, 27, 42, 43, 45, 47, 58, 78, 92, 118). All multistate characters were treated as unordered except the three that code positional change and are presumed to transform in an ordered fashion in development (8, 27, 43).

```
#NEXUS
Begin data;
        Dimensions ntax=16 nchar=102;
        Format symbols="0~9";
        Matrix
[Omeisaurus
    0100001010000000010000011??0??0001110111?100?40010000011000009911000100000000000?0
    09?09000000?10000?0??1
Jobaria
    010000111000000010000011000?00001110110010003000000?011000?0991000011000000000?0
    0900900001001000100?0
MACRONARIA
    0100001110000000100100110000000011100110100030000000011000009911000100000000000?0
    0900901001001000100000
Apatosaurus
    1001112011111001101010200100011220102111001410010001110000010310011110001001101
    1011110000101011000101
Suuwassea
    ??1????????????0?111000?00?0001?????02??1001400010001010000?001?100??????????1???
    ??00?10011111???????1
Diplodocus
    100111201111110011010102001000112201021110014?01100011100011103100111000010011 01
    1111110010011011000101
Brachytrachelopan
    ??????????????????????????????????????000??11??0011??111?1?110111?1??11???????
    ?????1????????????????
Dicraeosaurus
    101?????????00011110???011110112211021101012110000111111011110111100011100 1001
    1011?100?0011111000101
Amargasaurus
    ????????0???00011?110???0111111?????????00013110000?1??10100?130111????11???????
    ?????1???0??10????????
Limaysaurus
    ????????01?1001001091012101 0?11??????2??111??000?10?0000000099?1110000 1?00000110
    0911?11112000000110000
Nigersaurus
    00010110?1??001000?91012101101102212020 1111?300110110???0?009911?110111???1101??
    ??11?1?10201010?11101?
```

Rebbachisaurus
???????????????????????????????????2?2??111?????????????????99?11011?10????????
???????1?20?00??1?????
Cathartesaura
???0010?0?01?01199???????0????000110
09?????112????????????
Zapalasaurus
???000?0???????0????????????????????1?
??????????????001?00??
Histriasaurus
??101????????????????????1?10?????????????
??????????????????????
Spain_Rebbach.
??1????0011?11??????0????1??0??1???11000?
09?????????????0111?1?
;
END;

Temporal Range

The sources for temporal range data in Figure 4 is given below:

<u>Outgroups</u>		
Macronaria	Kimmeridgian-Maastrictian	
Jobaria	Neocomian	[7]
Omeisaurus	Oxfordian	[8]
<u>Ingroups</u>		
Cathartesaura	Turonian-Coniacian	[9]
Rebbachisaurus	Cenomanian	[7]
Limaysaurus	Aptian-early Turonian	[10]
Nigersaurus	Aptian-Albian	[7]
Histriasaurus	late Hauterivian-early Barremian	[11]
Zapalasaurus	Barremian-early Aptian	[11]
Burgos taxon	late Barremian-early Aptian	[12]
Amargasaurus	Barr-early Aptian	[10]
Dicraeosaurus	Kimmeridgian-Tithonian	[13]
Brachytrachylopan	Tithonian	[3]
Suuwassea	Tithonian	[14]
Diplodocus	Kimmeridgian, Tithonian	
Apatosaurus	Kimmeridgian, Tithonian	[15]

REFERENCES

1. Sereno PC (2007) Logical basis for morphological characters in phylogenetics.

Cladistics 23: 1-23.

2. Wilson JA (2002) Sauropod dinosaur phylogeny: critique and cladistic analysis. Zool J Linn Soc 136: 217-276.
3. Rauhut OWM, Remes K, Fechner R, Cladera G, Puerta P (2005) A remarkably short-necked sauropod dinosaur from the Late Jurassic of Patagonia. Nature 435: 670-672.
4. Salgado L, Bonaparte JF (1991) Un nuevo sauropodo Dicraeosauridae, *Amargasaurus casaui* gen. et sp. nov., de la fomación La Amarga, Neocomiano de la Provincia del Neuquen, Argentina. Ameghiniana 28: 333-346.
5. Calvo JO, Salgado L (1995) *Rebbachisaurus tessonei* sp. nov. a new Sauropoda from the Albian-Cenomanian of Argentina; new evidence on the origin of Diplodocidae. Gaia 11: 13-33.
6. Salgado L, Carvalho IS, Garrido AC (2006) *Zapalasaurus bonapartei*, a new sauropod dinosaur from La Amarga Formation (Lower Cretaceous), northwestern Patagonia, Neuquén Province, Argentina. Geobios 39: 695-707.
7. Sereno PC, Beck AL, Dutheil DB, Larsson HC, Lyon GH et al. (1999) Cretaceous sauropods from the Sahara and the uneven rate of skeletal evolution among dinosaurs. Science 286: 1342-1347.
8. He X, Wang C, Liu S, Zhou F, Liu T et al. (1998) A new sauropod dinosaur from the Early Jurassic in Gongxian County, South Sichuan. Acta Geologica Sichuan 18: 1-6.
9. Gallina PA, Apesteguía S (2005) *Cathartesaura anaerobica* gen. et sp. nov., a new rebbachisaurid (Dinosauria, Sauropoda) from the Huincul Formation (Upper Cretaceous), Río Negro, Argentina. Rev Mus Argentino Cienc Nat, n s 7: 153-166.

10. Leanza HA, Apestiguía S, Novas FE, Fuente MSdl (2004) Cretaceous terrestrial beds from the Neuquén Basin (Argentina) and their tetrapod assemblages. Cret Res 25: 61-87.
11. Dalla Vecchia FM (1998) Remains of Sauropoda (Reptilia, Saurischia) in the Lower Cretaceous (upper Hauterivianl/lower Baremian) limestones of SW Istria (Croatia). Geol Croatica 51: 105-134.
12. Pereda-Suberbiola X, Torcida F, Izquierdo LA, Huerta P, Montero D et al. (2003) First rebbachisaurid dinosaur (Sauropoda, Diplodocoidea) from the early Cretaceous of Spain: paleobiological implications. Bull Soc Géol France 174: 471-479.
13. Janensch W (1929) Die wirbelsäule der gattung *Dicraeosaurus*. Palaeontogr (Suppl 7) 2: 39-133.
14. Harris JD, Dodson P (2004) A new diplodocoid sauropod dinosaur from the Upper Jurassic Morrison Formation of Montana, USA. Acta Palaeontol Pol 49: 197-210.
15. Upchurch P, Martin J, Tomida Y, Barrett PM (2004) A new specimen of *Apatosaurus ajax* (Sauropoda: Diplodocidae) from the Morrison Formation (Upper Jurassic) of Wyoming, USA. Nat. Sci. Mus. Monogr. 26: 1–108.
